# Selective sodium halide over potassium halide binding and extraction by a heteroditopic halogen bonding [2]catenane†

**DOI:** 10.1039/d4sc03381g

**Published:** 2024-07-18

**Authors:** Hui Min Tay, Andrew Docker, Carol Hua, Paul D. Beer

**Affiliations:** a Chemistry Research Laboratory, Department of Chemistry, University of Oxford Mansfield Road Oxford OX1 3TA UK paul.beer@chem.ox.ac.uk; b Yusuf Hamied Department of Chemistry, University of Cambridge Lensfield Road Cambridge CB2 1EW UK; c School of Chemistry, The University of Melbourne Parkville Victoria 3010 Australia

## Abstract

The synthesis and ion-pair binding properties of a heteroditopic [2]catenane receptor exhibiting highly potent and selective recognition of sodium halide salts are described. The receptor design consists of a bidentate halogen bonding donor motif for anion binding, as well as a di(ethylene glycol)-derived cation binding pocket which dramatically enhances metal cation affinity over previously reported homo[2]catenane analogues. ^1^H NMR cation, anion and ion-pair binding studies reveal significant positive cooperativity between the cation and anion binding events in which cation pre-complexation to the catenane subsequently ‘switches-on’ anion binding. Notably, the heteroditopic catenane displayed impressive selectivity for sodium halide recognition over the corresponding potassium halides. We further demonstrate that the catenane is capable of extracting solid alkali metal salts into organic media. Crucially, the observed solution phase binding selectivity for sodium halides translates to superior functional extraction capabilities of these salts relative to potassium halides, overcoming the comparatively higher lattice enthalpies NaX > KX dictated by the smaller alkali metal sodium cation. This is further exemplified in competitive solid–liquid experiments which revealed the exclusive extraction of sodium halide salts from solid mixtures of sodium and potassium halide salts.

## Abstract 2

A heteroditopic [2]catenane is a potent ion-pair receptor for alkali metal halides. It preferentially binds sodium halides over potassium halides, which translates to selective extraction of solid sodium halide salts into organic solvents.

## Graphical abstract

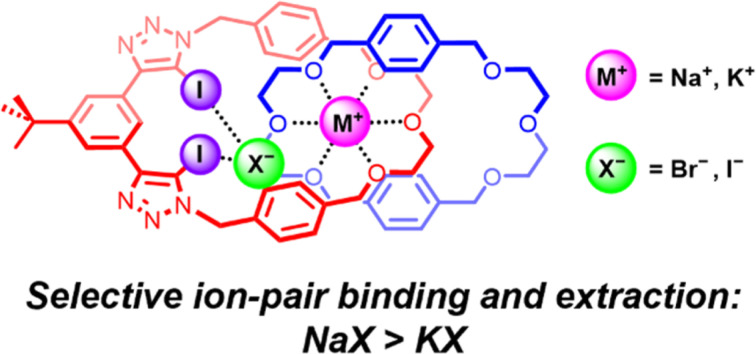

## Introduction

The field of supramolecular host–guest receptors based on mechanically interlocked molecules (MIMs) has expanded dramatically over the past two decades. A distinguishing feature of MIM-based receptors is the presence of topologically unique binding environments, which through judicious design, can be tuned to achieve size and shape complementarity towards a specific target guest.^1–9^ The resulting improvements in binding affinity and selectivity over non-interlocked receptor analogues have motivated the construction of numerous MIM-based cation^10–14^ and anion^15–20^ receptors over the years.

Heteroditopic receptors capable of the simultaneous binding of cations and anions, or ion-pair recognition, have been shown to exhibit dramatically augmented ion binding affinities over monotopic cation or anion receptors.^21–26^ These favourable binding properties have fuelled the rising prominence of ion-pair receptors in a myriad of applications including salt extraction/solubilisation,^27–37^ membrane transport^38,39^ and the recognition of biologically-relevant zwitterions.^40,41^

Despite these successes, the design of ion-pair receptors capable of distinguishing chemically-similar ions remains a persistent challenge. Of particular interest is the ability to discriminate between sodium and potassium ions, which are the primary cationic biological electrolytes and underpin many of the functions requisite for life.^42–44^ Their shared monocationic nature, spherical geometry and similar ionic radii make selective recognition of these ions a significant challenge in synthetic receptor systems,^45^ a difficulty underscored by the scarcity of heteroditopic ion-pair receptors capable of selectively binding sodium or potassium salts.^35,46,47^

The demonstrated efficacy of MIM-based receptors to engineer otherwise challenging ion selectivity profiles, alongside their modular nature which enables facile incorporation of multiple binding sites, makes them exquisitely suited to function as selective heteroditopic ion-pair receptors. It is therefore somewhat surprising that examples of MIM-based ion-pair receptors remain remarkably rare.^48–53^ Prompted by the envisaged utility of MIM-based ion-pair receptors, we previously reported a series of homo[2]catenanes capable of alkali metal halide binding *via* a di(ethylene glycol)-derived binding site for cation complexation alongside a bis(iodotriazole)benzene halogen bonding donor motif for anion binding.^54^ While this work highlighted the potential of exploiting the highly pre-organised binding cavities of catenanes to elicit the desired selectivity in sodium and potassium cation binding, the relatively moderate individual cation and anion affinities of these receptors precluded strong ion-pair binding.

**Fig. 1:**
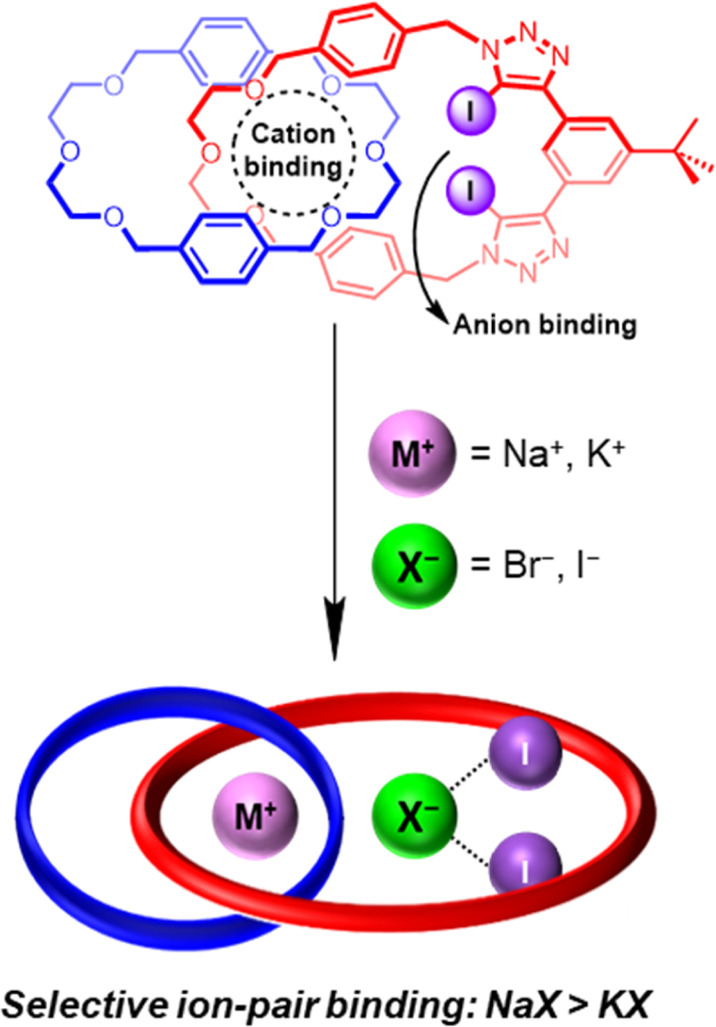
Schematic showing the chemical structure (top) and cartoon representation (bottom) of a hetero[2]catenane which functions as a heteroditopic ion-pair receptor capable of potent and selective binding of sodium halides.

Herein we report a heteroditopic hetero[2]catenane (Fig. 1) possessing a modified cation binding site serving to significantly increase its cation affinity, which in turn leads to a dramatic enhancement in the ion-pair binding affinity of the hetero[2]catenane relative to the homo[2]catenanes. Crucially, the use of di(ethylene glycol) linkers generates a cation binding pocket that exhibits a significantly closer size complementarity to the smaller sodium cation over the larger potassium cation. Notably, ^1^H NMR ion-pair binding studies revealed a preference for binding sodium halide salts over the corresponding potassium halide salts. Importantly, the observed preference for sodium halide binding in the solution phase manifests in superior and exclusive extraction of sodium halide salts, as demonstrated by a series of solid–liquid extraction studies.

## Results and discussion

### Synthesis and characterisation

**Scheme 1:**
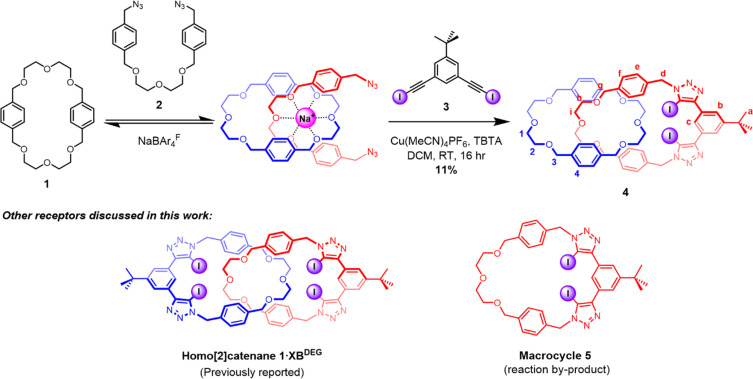
Synthesis of hetero[2]catenane 4 using a sodium cation template-directed strategy. The chemical structures of macrocycle 5 and a previously reported homo[2]catenane 1·XB^DEG^ are shown below.

It was envisaged that the target hetero[2]catenane could be prepared *via* a cation template-directed strategy^55–61^ in which a sodium ion directs the self-assembly of a pseudo[2]rotaxane complex between the crown ether like di(ethylene)glycol-functionalised macrocycle 1 and bis(azide) 2. Subjecting the threaded bis(azide) to a copper(i)-catalysed azide–alkyne cycloaddition (CuAAC) macrocyclisation reaction with a bis(iodoalkyne) would concurrently generate the second interlocked macrocycle and the halogen bonding anion binding site. To this end, macrocycle 1, NaBAr^F^ and bis(azide) 2 were pre-complexed in CH_2_Cl_2_ for 30 minutes, after which the bis(iodoalkyne) 3 was added, followed by dropwise addition of Cu(MeCN)_4_PF_6_ and tris[(1-benzyl-1*H*-1,2,3-triazol-4-yl)methyl]amine (TBTA). The reaction was allowed to stir at room temperature overnight, then washed with basic EDTA solution and water. Preparative thin-layer chromatographic purification afforded the target hetero[2]catenane 4 in 11% yield, which was characterised by ^1^H NMR, ^13^C NMR and high-resolution ESI-MS. It is postulated that the low yield is attributed to the competing macrocyclisation reaction to afford non-interlocked 5, which was isolated as the major by-product of the reaction (Scheme 1).

**Fig. 2:**
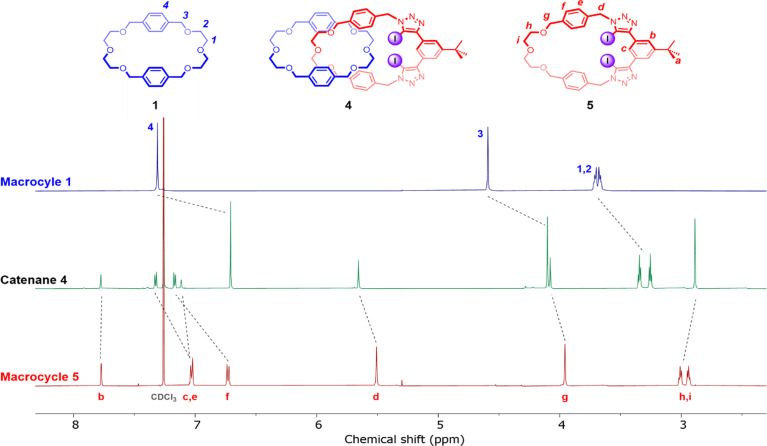
Stacked ^1^H NMR spectra of macrocycle 1 (top), [2]catenane 4 (middle) and XB macrocycle 5 (bottom) (500 MHz, CDCl_3_, 298 K).

Comparing the ^1^H NMR spectrum of [2]catenane 4 in CDCl_3_ solution to those of its constituent macrocycles 1 and 5 revealed diagnostic peak shifts consistent with an interlocked topology (Fig. 2). The proton resonances arising from macrocycle 1 underwent significant upfield shifts upon formation of the [2]catenane, attributed to the shielding effect associated with the enforced proximity of these environments to the shielding ring currents of the xylene spacers in 5. No proton signal desymmetrisation of the macrocycles was observed, indicating that under ambient conditions in CDCl_3_ the relative co-conformational circumrotation of the constituent macrocycles is facile, *i.e.* fast relative to the NMR timescale. Significant perturbations were also observed in the signals arising from 5, most notably upfield shifts and coalescence of protons H_h_ and H_i_, indicating that the ethylene glycol region of 5 is threaded through the cavity of 1.

### ^1^H NMR binding studies

To investigate the potential of the hetero[2]catenane 4 to function as an ion-pair receptor for alkali metal halide salts, ^1^H NMR binding studies with sodium and potassium tetrakis[3,5-bis(trifluoromethyl)phenyl]borate (M^I^BAr^F^_4_, where M^I^ = Na^+^, K^+^) were initially undertaken to establish the cation binding ability of 4 in 1 : 1 CD_3_CN/CDCl_3_.

**Fig. 3:**
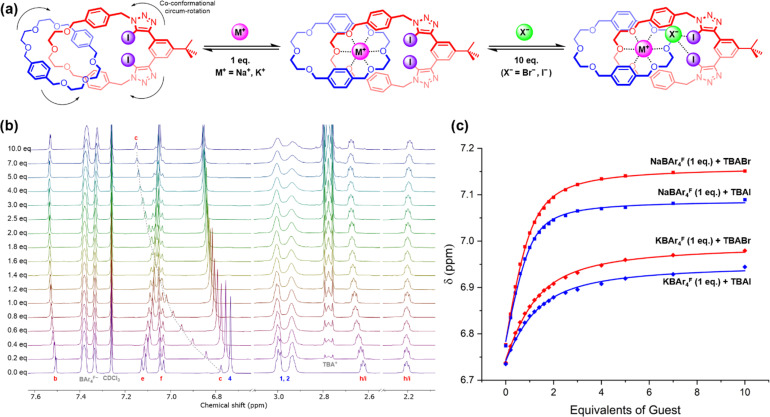
Ion-pair binding studies of hetero[2]catenane 4, including: (a) proposed ion-pair binding mode, showing the pre-complexation of the cation in the di(ethylene glycol)-derived binding cavity, followed by anion binding at the XB donor group. (b) Truncated stacked ^1^H NMR spectra of 4 upon progressive addition of TBABr in the presence of 1 eq. NaBAr^F^_4_. (c) Binding isotherms of 4, showing changes in the chemical shift of H_c_ with increasing equivalents of TBAX salts (X = Br, I) in the presence of 1 eq. MBAr^F^_4_ (M = Na, K). For (b) and (c), [receptor] = 1.0 mM. Solvent = 3 : 1 CD_3_CN/CDCl_3_ (v/v). *T* = 298 K.

Upon addition of NaBAr^F^_4_ and KBAr^F^_4_ to 4, large perturbations were observed in the ethylene glycol proton resonances of the two constituent macrocycles, as well as the proximal benzylic singlets H_g_ and H_3_, consistent with convergent coordination of the alkali metal cation by the polyether oxygen atoms of both macrocycles (Fig. S7–S8†). A substantial downfield shift of the xylene protons H_4_ from macrocycle 1 was also observed, possibly indicative of a co-conformational rearrangement that rotates these aromatic groups away from the macrocyclic cavity of 5 to accommodate the cation in the binding site, a postulated structure of which is shown in Fig. 3a.

The binding isotherms generated by monitoring the upfield perturbations of ethylene glycol proton H_i_ rapidly approached saturation upon the addition of 1 equiv. of the cation (Fig. S36†). Fitting the isotherms to a 1 : 1 host–guest stoichiometric binding model using Bindfit analysis^62^ gave cation association constants of approximately 58 000 M^−1^ and 28 000 M^−1^ for Na^+^ and K^+^ respectively, indicating preferential sodium over potassium binding, although the high fitting errors (10–20%) precluded an accurate determination of the Na^+^ > K^+^ binding selectivity (Table 1). Importantly, this represents a significant enhancement (>10-fold) in cation affinity relative to previously reported homo[2]catenane 1·XB^DEG^ (*K*_a_ (Na^+^) = 756(11) M^−1^, *K*_a_ (K^+^) = 284(2) M^−1^), which possesses a cation binding site comprised of two ethylene glycol linkers from the corresponding non-interlocked constituent macrocycles 5. The symmetric nature of macrocycle 1 would conceivably allow [2]catenane 4 to adopt a larger number of co-conformations in which convergent cation coordination by the polyether regions of its two constituent macrocycles can be achieved. Crucially, analogous cation binding studies conducted on the non-interlocked macrocyclic components 1 and 5 in 1 : 1 CD_3_CN/CDCl_3_ (Fig. S32–S36†) revealed no significant cation affinity, highlighting the necessity of the interlocked binding cavity for cation binding.

**Table 1:** Cation association constants (*K*_a_/M^−1^) for [2]catenanes determined by ^1^H NMR titrations^a^

Solvent	1 : 1 CD_3_CN/CDCl_3_	3 : 1 CD_3_CN/CDCl_3_	
Receptor	Homo[2]catenane 1·XB^DEG^	Hetero[2]catenane 4	
Na^+^	756(11)^b^	58 000 (6000)	20 000(750)
K^+^	284(2)^b^	27 000 (5000)	3737(91)

a *K*_a_ values calculated using Bindfit with a 1 : 1 host–guest binding model. Errors are shown in parantheses. All cations added as BAr_4_^F−^ salts. *T* = 298 K. [Receptor] = 1.0 mM.

b Reported in ref. 54.

To further investigate the relative affinities of [2]catenane 4 for sodium and potassium ions, the ^1^H NMR cation binding studies were repeated in the more competitive solvent mixture 3 : 1 CD_3_CN/CDCl_3_ (Fig. S9–S10 and S37†), wherein cation binding affinities should be attenuated relative to the 1 : 1 CD_3_CN/CDCl_3_ solvent system. In this solvent system the *K*_a_(Na^+^) value was determined to be approximately 20 000 M^−1^, which is significantly higher (>5-fold) than the corresponding *K*_a_(K^+^) value (Table 1). This indicates that [2]catenane 4 preferentially binds sodium over potassium, presumably due to more optimal size complementarity between the di(ethylene glycol)-derived binding site and the smaller sodium cation.^57^

The anion binding affinity of [2]catenane 4 was investigated *via* analogous ^1^H NMR titrations with tetrabutylammonium halide salts (TBAX, where X = Cl, Br, I). In 1 : 1 CD_3_CN/CDCl_3_, the addition of aliquots of TBAX surprisingly resulted in significant peak broadening and merging (Fig. S11–S13†). Consequently, the halide association constants of 4 could not be reliably determined in this solvent system. In contrast, the ^1^H NMR signals of the [2]catenane host remained sharp in 3 : 1 CD_3_CN/CDCl_3_, which is attributed to the greater solubility of the [2]catenane–anion complex in this solvent system. A significant downfield perturbation of the internal benzene signal H_c_ was observed, suggesting that halide anion binding occurs *via* XB interactions with the bis(iodotriazole)benzene donor motif (Fig. S14–S16†). Bindfit^62^ analysis of the resulting binding isotherms (Fig. S38†) unexpectedly revealed that 4 exhibits approximately 2-fold higher halide association constants than its constituent XB macrocycle 5 (Table 2). Whilst initially surprising, since the mechanical bond between crown ether-like macrocycle 1 to macrocycle 5 to form [2]catenane 4 ostensibly introduces no additional anion binding groups, we tentatively postulate this enhanced halide affinity is attributable to the formation of ancillary CH⋯X^−^ interactions involving the aromatic spacers of macrocycle 1, which underwent downfield perturbations upon addition of TBA halide salts, and/or co-conformational restraints imposed on the XB anion binding motif imposed by the mechanical bond.^63^

**Table 2:** Anion association constants (*K*_a_/M^−1^) for [2]catenane 4 and XB macrocycle 5 determined by ^1^H NMR titrations^a^

Receptor	Hetero[2]catenane 4	Macrocycle 5
Cl^−^	419(34)	212(2)
Br^−^	506(44)	261(2)
I^−^	437(29)	277(2)

a *K*_a_ values calculated using Bindfit with a 1 : 1 host–guest binding model. Errors are shown in parantheses. All anions added as TBA^+^ salts. Solvent = 3 : 1 CD_3_CN/CDCl_3_. *T* = 298 K. [Receptor] = 1.0 mM.

Having established that the binding affinity of 4 for alkali metal cations is sufficient to ensure a high degree of saturation of the receptor in the presence of 1 equiv. of the cation in 1 : 1 CD_3_CN/CDCl_3_, ^1^H NMR ion-pair binding studies were conducted by adding aliquots of TBAX salts (X = Cl, Br, I) to a 1 mM solution of 4 containing an equimolar amount of MBAr^F^_4_ (M = Na, K) (Fig. 3a).

Upon addition of Br^−^ and I^−^, prominent downfield shifts of the internal benzene proton H_c_ were observed, which suggest that anion binding remains primarily mediated by XB interactions (Fig. S18, S19, S21 and S22†). Importantly, the addition of bromide and iodide causes little to no perturbation of the ethylene glycol proton signals, indicating that the cation binding sites of the catenane remain saturated throughout the titration. In contrast, addition of chloride to the receptor–cation complex induces chemical shift perturbations consistent with external ion-pairing and salt recombination (Fig. S17 and S20†), indicating that despite the high cation affinities of 4, its ion-pair binding ability remains insufficient to overcome the high lattice enthalpies of NaCl and KCl.

In an analogous manner to the anion binding studies, binding isotherms for Br^−^ and I^−^ were constructed by monitoring the downfield shift of H_c_ and subsequently fitted to a 1 : 1 host–guest binding model (Fig. S39 and S40†) to determine apparent anion association constants (*K*_app_) of the monocationic catenane–Na^+^ complexes, assuming near-quantitative pre-complexation of the metal cation to the catenane host. The calculated *K*_app_ values were in range 10 000–15 000 M^−1^ in 1 : 1 CD_3_CN/CDCl_3_, demonstrating a dramatic enhancement in the anion binding affinity of [2]catenane 4 in the presence of a co-bound sodium cation. The corresponding *K*_app_ values of the catenane–K^+^ complex were found to be significantly lower (Table 3), suggesting a binding preference for sodium halide over potassium halide salts by the [2]catenane receptor. Importantly, the qualitatively similar chemical shift perturbations observed in the NaX and KX titrations, namely a downfield shift of H_c_ and H_4_ upon addition of Br^−^ and I^−^ with no significant changes in the ethylene glycol proton signals, indicate that ion-pair binding of KBr and KI by 4 nonetheless remains the dominant process, outcompeting the salt recombination equilibrium. These studies crucially demonstrate strong positive cooperativity between the cation and anion binding events which enables the heteroditopic [2]catenane to act as a potent ion-pair receptor.

**Table 3:** Apparent anion association constants (*K*_app_/M^−1^) for catenane–cation complexes of 4 determined by ^1^H NMR titrations^a^

Solvent	Cation	Anion	*K*_app_ (M^−1^)
1 : 1 CD_3_CN/CDCl_3_	Na^+^	Cl^−^	Salt recombination
		Br^−^	10 300(1700)
		I^−^	15 200(3000)
	K^+^	Cl^−^	Salt recombination
		Br^−^	3178(269)
		I^−^	2357(268)
3 : 1 CD_3_CN/CDCl_3_	Na^+^	Cl^−^	Salt recombination
		Br^−^	4179(204)
		I^−^	5392(522)
	K^+^	Cl^−^	Salt recombination
		Br^−^	1480(69)
		I^−^	1243(96)

a *K*_app_ values calculated using Bindfit with a 1 : 1 host–guest binding model. Errors are shown in parantheses. All cations added as BAr_4_^F−^ salts and anions as TBA^+^ salts. *T* = 298 K. [Receptor] = [MBAr^F^_4_] = 1.0 mM.

In order to quantitatively measure the apparent anion binding constants of the catenane–cation complexes, the ion-pair binding studies were repeated in the more competitive solvent system 3 : 1 CD_3_CN/CDCl_3_ (Fig. 3b and c). Crucially, the *K*_app_ values determined for the sodium halide salts were 2 to 5-fold higher than the analogous potassium salts (Table 3), indicating that the size-driven selectivity of the [2]catenane 4 for sodium over potassium cations translates to its ion-pair recognition properties.

### Solid-state X-ray crystallographic studies

**Fig. 4:**
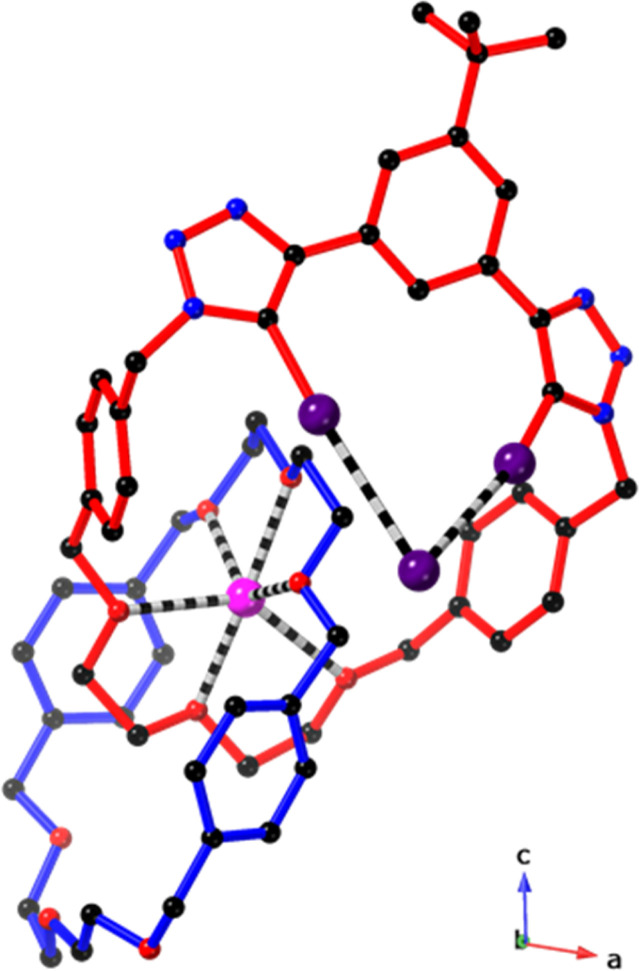
Solid-state structure of 4 bound to an NaI ion-pair. Hydrogen atoms have been omitted for clarity. Atom colours are as follows: black (carbon), blue (nitrogen), red (oxygen), purple (iodine), pink (sodium).

The proposed ion-pair binding mode of 4 was further corroborated by solid-state structural analysis.‡‡Low temperature single crystals X-ray diffraction data were collected using an Oxford Diffraction Supernova X-ray diffractometer and reduced using CrysAlisPro. The structures were solved using SHELXS [G. Sheldrick, *Acta Crystallogr., Sect. A: Found. Adv.*, 2015, **71**, 3–8] and refined using CRYSTALS [P. W. Betteridge, J. R. Carruthers, R. I. Cooper, K. Prout and D. J. Watkin, *J. Appl. Crystallogr.*, 2003, **36**, 1487; R. I. Cooper, A. L. Thompson and D. J. Watkin, *J. Appl. Crystallogr.*, 2010, **43**, 1100–1107] and SHELXL [G. Sheldrick, *Acta Crystallogr., Sect. C: Struct. Chem.*, 2015, **71**, 3–8]. Full details are included in the accompanying ESI (CIF). The data can be obtained from the joint Cambridge Crystallographic Data Centre and Fachinformationszentrum Karlsruhe Access Structures service https://www.ccdc.cam.ac.uk/structures with deposition number 2348225. Slow evaporation of a 1 : 1 CD_3_CN/CDCl_3_ solution containing hetero[2]catenane 4 in the presence of NaBAr^F^_4_ and excess TBAI afforded single crystals suitable for analysis by single crystal X-ray diffraction. The solid-state crystal structure revealed a 1 : 1 complex of catenane 4 bound to an NaI ion-pair, in which the guest ions are bound in a host-separated ion-pair binding mode consistent with solution-phase NMR studies (Fig. 4). Notably, the polyether regions of the two interlocked macrocyclic components 1 and 5 are arranged in an orthogonal geometry to form a cation binding pocket. The sodium cation resides within this binding pocket where it is convergently bound by three oxygen atoms each from 1 and 5 to form a six-coordinate distorted octahedral metal centre. In contrast, the iodide anion is located on the periphery of the macrocycle 5 where it forms XB interactions with the bis(iodotriazole)benzene motif, as shown by the I⋯I^−^ distances (3.4597(7)–3.4820(6) Å, which is 87–98% of the sum of the van der Waals radii of the two atoms). The absence of ancillary CH–X^−^ interactions between macrocycle 1 and the iodide guest in 4·NaI may be due to steric constraints imposed by the formation of a cation binding pocket, which presumably restricts the inter-ring circum-rotation of the catenane.

### Solid–liquid extraction studies

The promising ion-pair binding properties of hetero[2]catenane 4 prompted an investigation into its ability to act as an alkali halide salt extractant. To this end, solid–liquid extraction (SLE) studies were undertaken by sonicating a 1 mM CDCl_3_ solution of 4 in the presence of a fixed excess mass quantity of solid MX (M = Na, K; X = Cl^−^, Br^−^, I^−^).

**Fig. 5:**
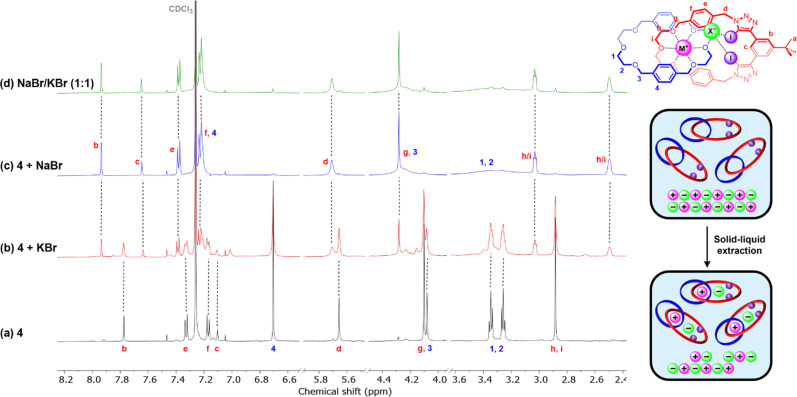
Solid–liquid extraction studies of alkali metal bromide salts, showing truncated ^1^H NMR spectra of: (a) free catenane receptor 4, and post-extraction spectra of 4 following treatment with (b) 10 mg KBr; (c) 10 mg NaBr; (d) 5 mg NaBr and 5 mg KBr (CDCl_3_, 298 K, 500 MHz). The inset on the right shows the structure of 4 bound to an MX ion-pair (top) and a schematic illustrating the SLE experiment (bottom).

Large perturbations were observed in the ^1^H NMR spectra of 4 post-treatment with NaBr and NaI (Fig. 5c and S45c†). In particular, a dramatic upfield shift of internal benzene proton H_c_ was observed as well as desymmetrisation of the ethylene glycol protons H_h/I_, which are respectively consistent with anion and cation binding by the [2]catenane. These results indicate complete stoichiometric extraction of both NaX salts, with the post-extraction solution containing only the [2]catenane as the ion-pair bound complex.

In contrast, treatment of 4 with KBr and KI resulted in more complex ^1^H NMR spectra which upon closer inspection revealed two sets of proton signals corresponding to two distinct catenane species. In the case of KBr, shown in Fig. 5b, the major set exhibits chemical shifts similar to those of the unbound receptor while the minor set resembles the spectrum of the NaBr-bound catenane complexes. These observations suggest incomplete KBr extraction with the bound and unbound catenane complexes in slow exchange. Integration of these signals estimated that 43% of the hetero[2]catenane in solution was present as the ion-pair bound complex.

No significant signal perturbations were observed upon treatment of 4 with NaCl or KCl (Fig. S47†), which was attributed to the high lattice enthalpy of alkali metal chloride salts outcompeting ion-pair binding to 4, and is consistent with the observation of salt recombination during titration studies of the catenane–metal complexes with TBACl.

Motivated by the observed superior performance of 4 for sodium bromide/iodide extraction over the corresponding potassium salts, we sought to further investigate the extraction preferences of the catenane receptor *via* a competitive NaX/KX solid–liquid extraction experiment. To this end, in an analogous procedure to the above-described experiments, a 1 mM CDCl_3_ solution of 4 was exposed to a mixture of NaBr/KBr or NaI/KI present in excess. In both cases, this resulted in post-extraction spectra identical to those obtained from treatment of the receptor solely with the corresponding sodium halide salt (Fig. 5d and S45d†). Crucially, this provides promising evidence that 4 is capable of preferential sodium halide extraction over the corresponding potassium halide salts. Importantly, this highlights that despite the higher lattice enthalpies of sodium halide over potassium halide salts, dictated by the smaller alkali metal sodium cation, the mechanical bond effect enables size-based discrimination and thereby achieves selective ion-pair recognition and extraction.

## Conclusions

In summary, a heteroditopic [2]catenane ion-pair host system containing an interchain component di(ethylene glycol)-derived cation binding site and a bidentate halogen bonding anion binding site was synthesised *via* a sodium cation template-directed approach. ^1^H NMR cation binding studies revealed a dramatic enhancement in sodium and potassium alkali metal cation affinity relative to a previously reported series of homo[2]catenanes, as well as considerable sodium over potassium selectivity. The alkali metal halide (MX, X = Br, I) ion-pair binding properties of the catenane were probed by ^1^H NMR studies and X-ray crystallography, both of which were consistent with the proposed separated ion-pair binding mode. Ion-pair titrations showed strong binding of alkali metal halide salts with significant cooperativity between the cation and anion binding events, reflected in a significant ‘switch-on’ of the catenane's anion affinity upon pre-complexation with a metal cation. Importantly, the catenane was found to bind sodium halide salts 3–5 times more strongly than the corresponding potassium halide salts. Solid–liquid extraction studies highlighted the capability of the catenane to function as an efficient alkali metal halide salt extractant, in which the observed selectivity preference for sodium halide over potassium halide ion-pair binding in solution-state NMR studies impressively translated to superior and exclusive extraction of sodium halide salts from a competitive solid mixture of NaX/KX salts. The potent ion-pair binding capabilities of this heterocatenane receptor, and importantly its ability to discriminate between sodium halide and potassium halide salts, demonstrates the efficacy of exploiting the mechanical bond effect to elicit improved binding affinity and selectivity, which will serve to inform the design of future heteroditopic ion-pair receptors.

## Data availability

The data supporting this article have been included as part of the ESI.† Crystallographic data for compound 4·NaI has been deposited at the CCDC under 2348225 and can be obtained from https://www.ccdc.cam.ac.uk/structures.

## Author contributions

P. D. B. conceived the project. H. M. T. conducted the experimental studies. C. H. conducted the X-ray crystallographic analysis. All authors contributed to the writing and editing of the manuscript and agreed on the final version.

## Conflicts of interest

There are no conflicts to declare.

## Supplementary Material

SC-015-D4SC03381G-s001

SC-015-D4SC03381G-s002
