# Supplementary material for: Selective sodium halide over potassium halide binding and extraction by a heteroditopic halogen bonding [2]catenane
Source: Chem Sci. 2024 Jul 18;15(32):13074–81. doi: 10.1039/d4sc03381g (PMC11322978; doi:10.1039/d4sc03381g)
Supplement: SC-015-D4SC03381G-s001 [file SC-015-D4SC03381G-s001.pdf]

Electronic Supplementary Material (ESI) for Chemical Science.
This journal is © The Royal Society of Chemistry 2024

Supporting Information for

Selective Sodium Halide over Potassium Halide Binding and Extraction by a Heteroditopic Halogen Bonding [2]Catenane

Hui Min Tay, Andrew Docker, Carol Hua and Paul D. Beer

Contents

Materials and Methods 2
Synthesis and Characterisation of Novel Compounds 3
^{1}H NMR binding studies 8
X-ray Crystallographic Studies 27
Solid-liquid Extractions 28
References 31

Materials and Methods

General

All solvents and reagents were purchased from commercial suppliers and used as received unless otherwise stated. Dry solvents were obtained by purging with nitrogen and then passing through an MBraun MPSP-800 column. H_2O was de-ionized and micro filtered using a Milli-Q® Millipore machine. Column chromatography was carried out on Merck® silica gel 60 under a positive pressure of nitrogen. Routine NMR spectra were recorded on either a Bruker AVIII 400, Bruker AVIII 500 or a Bruker AVIII 600 spectrometer with ^1H NMR titrations recorded on a Bruker AVIII 500 spectrometer. TBA salts were stored in a vacuum desiccator containing phosphorus pentoxide prior to use. Where mixtures of solvents were used, ratios are reported by volume. Chemical shifts are quoted in parts per million relative to the residual solvent peak. Mass spectra were recorded on a Bruker µTOF spectrometer. Triethylamine was distilled from and stored over potassium hydroxide. Tris[(1-benzyl-1H-1,2,3- triazol-4-yl)methyl]amine (TBTA),[1] macrocycle **1**[2], bis(azide) **2**[3] and 1-(tert-butyl)-3,5-bis(iodoethynyl)benzene **3**[4] were prepared according to previous literature reports. Macrocycle **5** was isolated as a by-product during the synthesis of **4** and its spectral analysis is in line with previously reported data.[3]

Synthesis and Characterisation of Novel Compounds

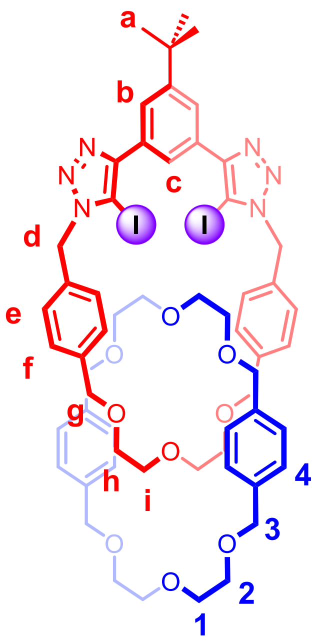

XB hetero[2]catenane (4). Macrocycle **1** (30.0 mg, 0.072 mmol) and $NaBAr_4^F$ (63.8 mg, 0.072 mmol) were dissolved in dry, degassed CH_2Cl_2 (2.0 mL) and stirred for 30 minutes at room temperature. A solution of bis-azide **2** (28.6 mg, 0.072 mmol) in CH_2Cl_2 (1.0 mL) was added and the mixture stirred for a further 30 minutes. A solution of bis(iodoalkyne) **3** (31.3 mg, 0.072 mmol) in CH_2Cl_2 (1.0 mL) was added, followed by a dropwise addition of a premixed solution of $[Cu(CH_3CN)_4]PF_6$ (13.4 mg, 0.036 mmol) and TBTA (19.1 mg, 0.024 mmol) in CH_2Cl_2 (1.0 mL). The reaction mixture was stirred at room temperature in the dark for 20 hours, then was diluted with CH_2Cl_2 (25 mL). The organic layer was washed with EDTA/NH_4OH (2 × 20 mL) and H_2O (2 × 20 mL), dried over $MgSO_4$, filtered and concentrated under vacuum. The crude was purified by iterative preparative TLC in 7:3 EtOAc/CH_2Cl_2, followed by 4:96 MeOH/DCM to afford the target catenane **4** as a white solid (10 mg, 11%).

^{1}H NMR (600 MHz, $CDCl_3$) δ (ppm) 7.78 (d, J = 1.6 Hz, 2H, H_b), 7.33 (d, J = 7.9 Hz, 4H, H_e), 7.17 (d, J = 7.9 Hz, 4H, H_f), 7.12 (d, J = 1.6 Hz, 1H, H_c), 6.71 (s, 8H, H_4), 5.66 (s, 4H, H_d), 4.10 (s, 8H, H_3), 4.08 (s, 4H, H_g), 3.34 (t, J = 5.7 Hz, 8H, $H_{1/2}$), 3.26 (t, J = 5.7 Hz, 8H, $H_{1/2}$), 2.89 (s, 8H, H_h, H_i), 1.40 (s, 9H, H_a).

^{13}C NMR (151 MHz, $CDCl_3$) δ (ppm) 152.22, 152.07, 139.80, 137.14, 133.48, 130.21, 129.56, 128.98, 128.73, 128.07, 125.98, 125.31, 73.19, 72.15, 70.40, 69.52, 68.94, 68.43, 54.47, 35.20, 31.48, 29.85.

HRMS (ESI +ve) m/z: 1247.3160 ($[M+H]^+$, $C_{58}H_{69}O_9N_6I_2$ requires 1247.3210).

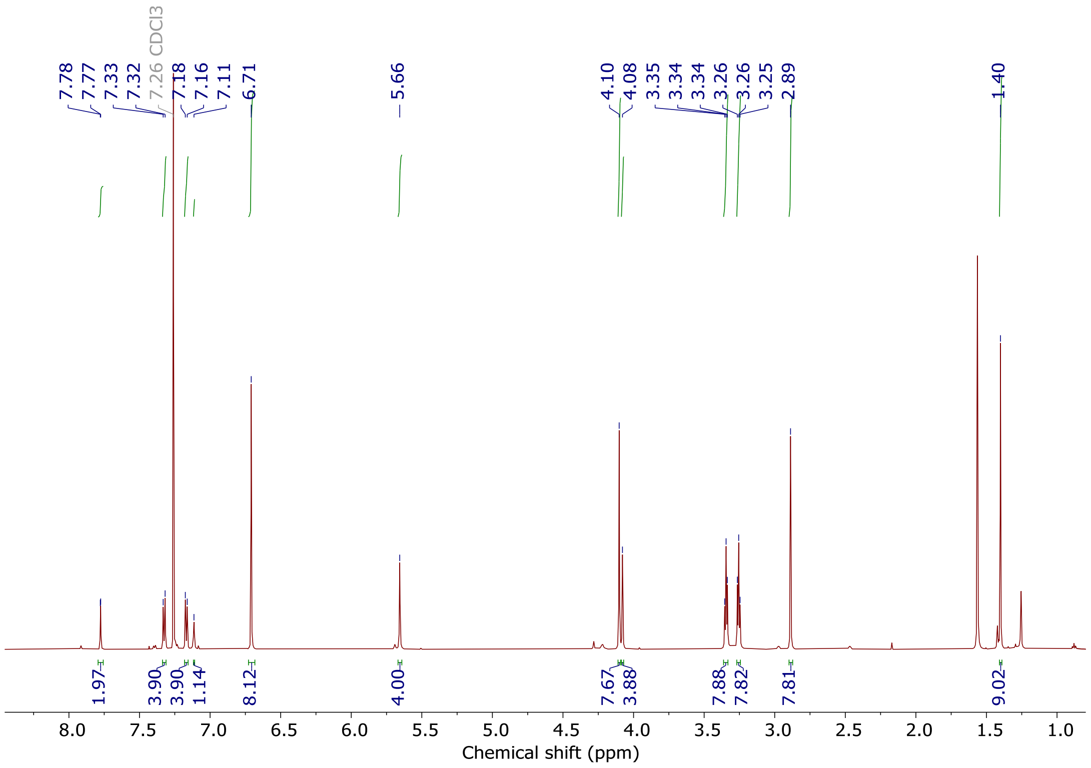

*Figure S1. ^{1}H-NMR spectrum of hetero[2]catenane **4** (600 MHz, $CDCl_3$, 298 K)*

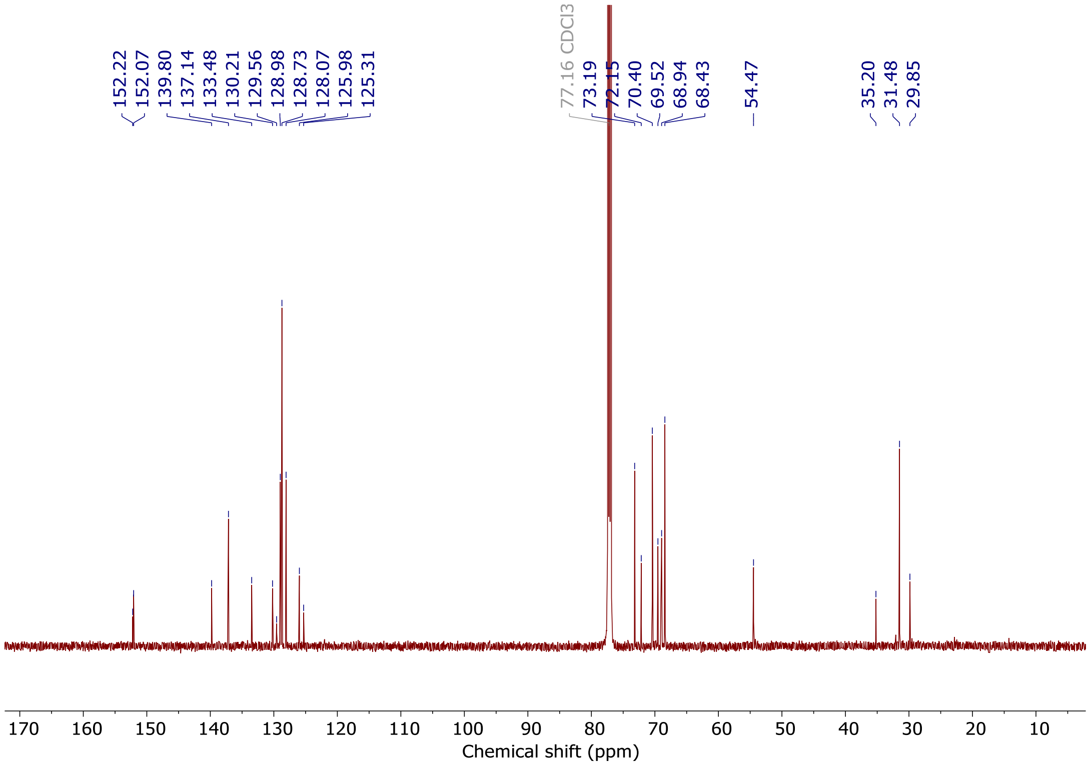

*Figure S2. ^{13}C-NMR spectrum of hetero[2]catenane **4** (151 MHz, $CDCl_3$, 298 K)*

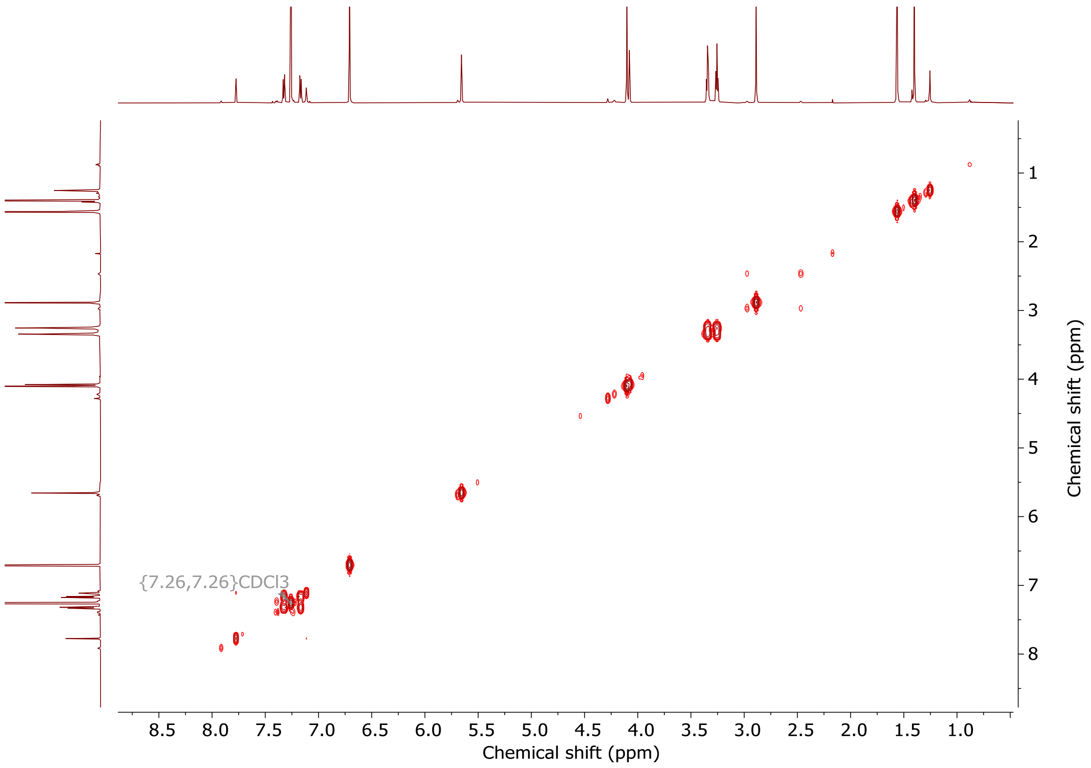

Figure S3. ^1H-^1H 2D COSY NMR spectrum of hetero[2]catenane ***4*** *(600 MHz, $CDCl_3$, 298 K)*

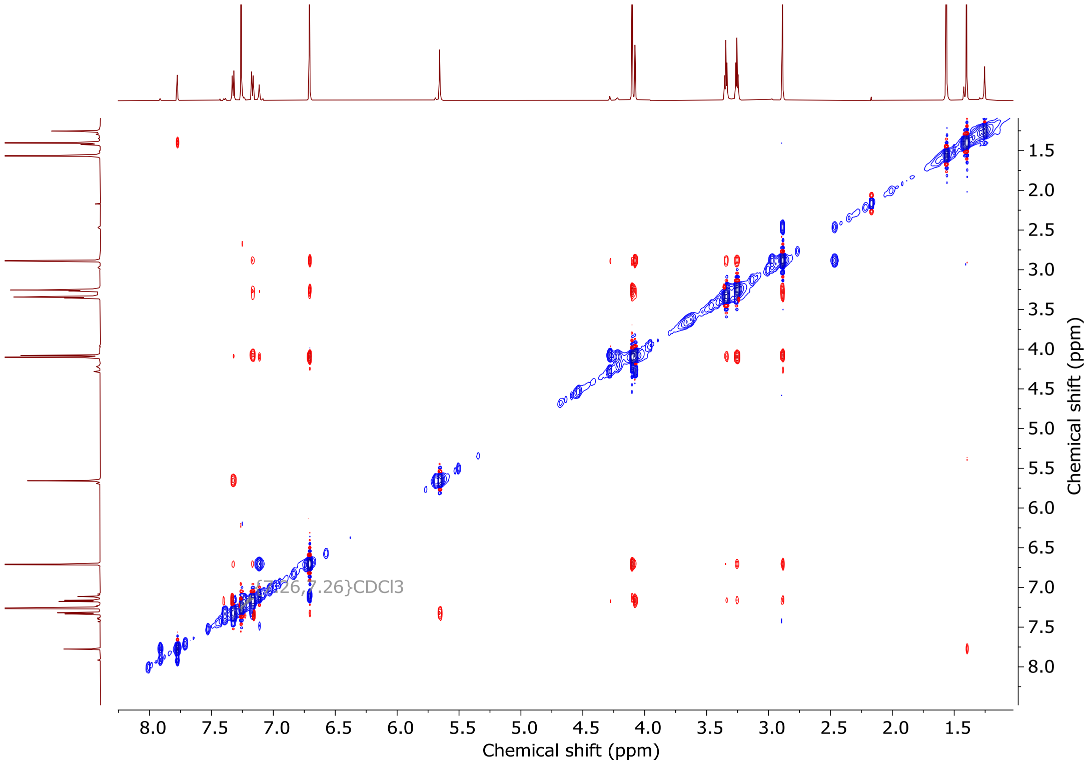

Figure S4. ^1H-^1H 2D ROESY NMR spectrum of hetero[2]catenane ***4*** *(600 MHz, $CDCl_3$, 298 K)*

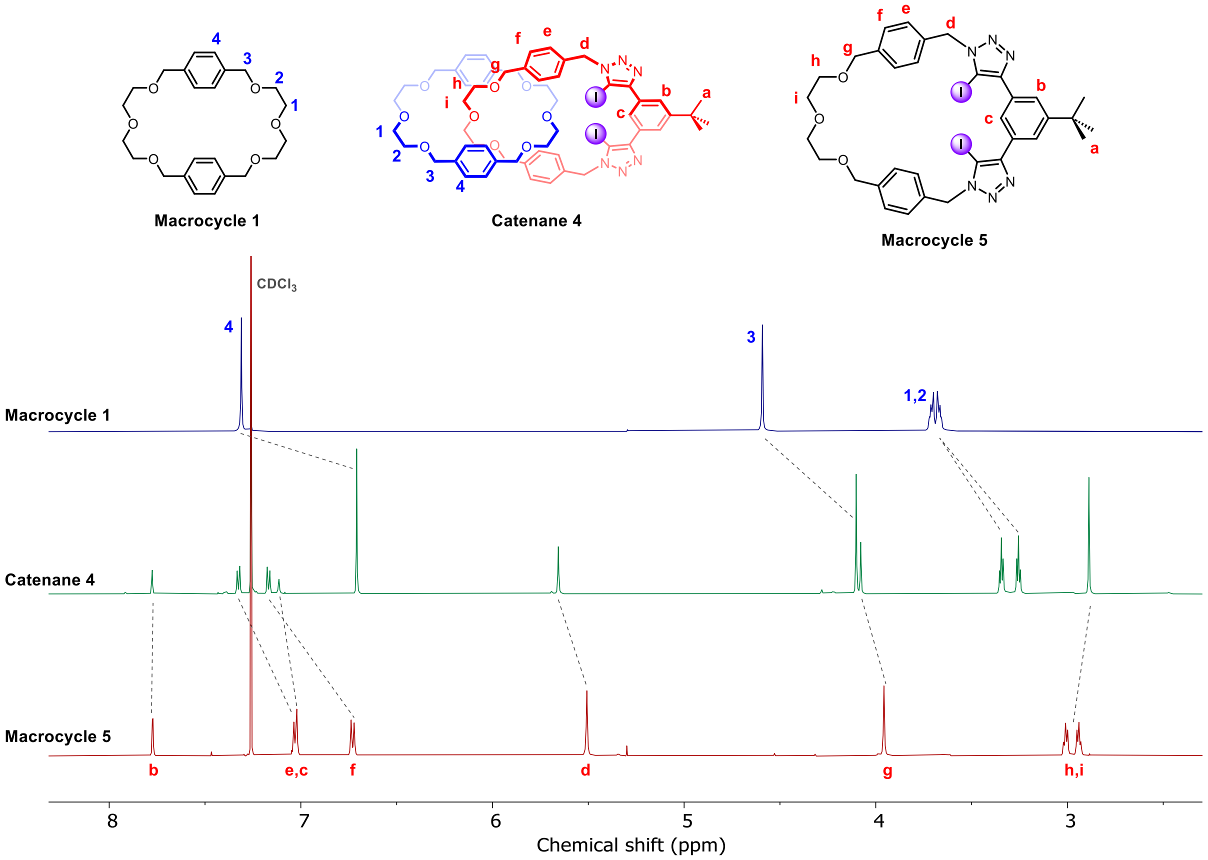

*Figure S5. Overlaid ^{1}H NMR spectra of macrocycle **1**, hetero[2]catenane **4** and XB macrocycle **5** (500 MHz, $CDCl_3$, 298 K).*

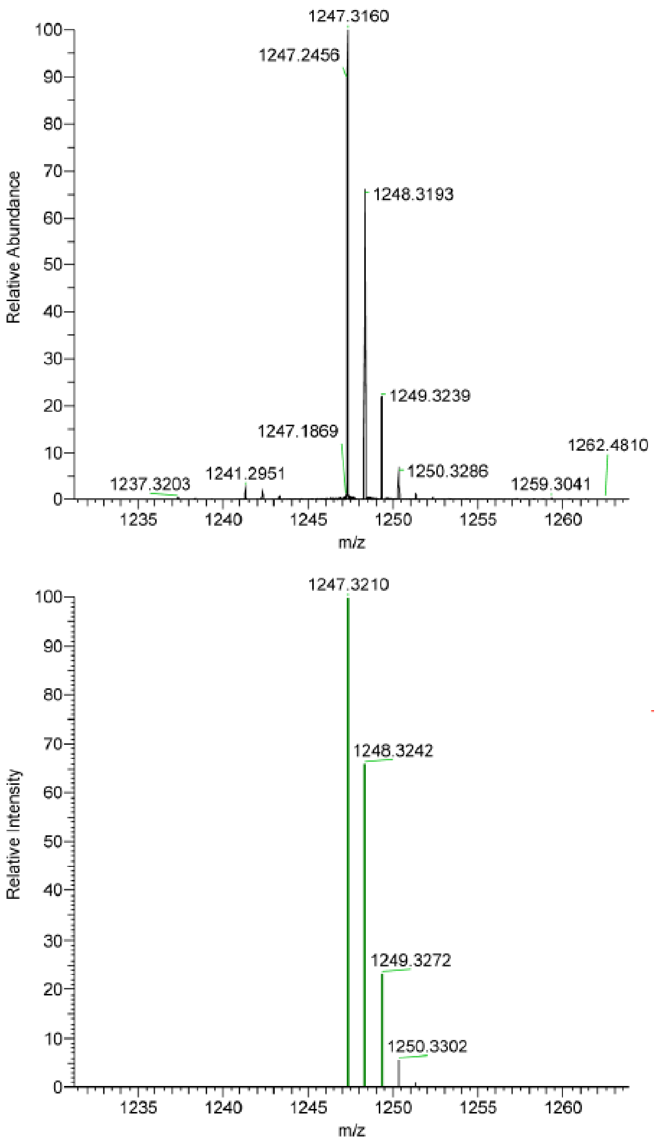

Figure S6. High-resolution mass spectrum (ESI +ve) of hetero[2]catenane ***4*** *(top: experimental; bottom: theoretical).*

^{1}H NMR binding studies

General procedure

All ^{1}H NMR titration experiments were performed on a Bruker AVIII 500 MHz spectrometer at 298 K. In a typical cation or anion titration, a 1.0 mM solution of the neutral receptor was prepared in 1:1 or 1:3 $CDCl_3/CD_3CN$. In an ion-pair titration, an equimolar amount of the [2]catenane and M^IBAr^F, each present at 1.0 mM concentration, was dissolved in 1:1 or 1:3 $CDCl_3/CD_3CN$. The solution was sonicated for 20 min to form the metal-catenane complex. A 50 mM solution of the guest ion, either M^IBAr^F (M = Na, K) or TBAX (X = Cl, Br, I), was added in aliquots to the solution containing the receptor, where 1.0 equivalent of the salt added corresponds to 10.0 μL of the salt solution. 17 spectra were recorded, corresponding to 0.0, 0.2, 0.4, 0.6, 0.8, 1.0, 1.2, 1.4, 1.6, 1.8, 2.0, 2.5, 3.0, 4.0, 5.0, 7.0, 10.0 equivalents of the added guest ion. The binding of cations and anions to all receptors were found to be fast on the NMR timescale. For cation titrations, the chemical shifts of multiple peaks around the cation binding site (H_g, $H_{h/i}$) were monitored and used for subsequent fitting. For anion and ion-pair titrations, the chemical shift of the internal benzene proton H_c was used. The values of the observed chemical shift(s) and concentration of guest at each titration point were entered into the Bindfit software alongside initial estimates of the binding constants and limiting chemical shifts. These parameters were refined using nonlinear least-squares analyses to obtain the best fit between empirical and calculated chemical shifts based on five host-guest binding models (1:1, 1:2 Full, 1:2 Non-cooperative, 1:2 Additive and 1:2 Non-statistical). The input parameters were varied until convergence of the best fit values of the binding constants was attained.

^{1}H NMR titration spectra

Cation titrations

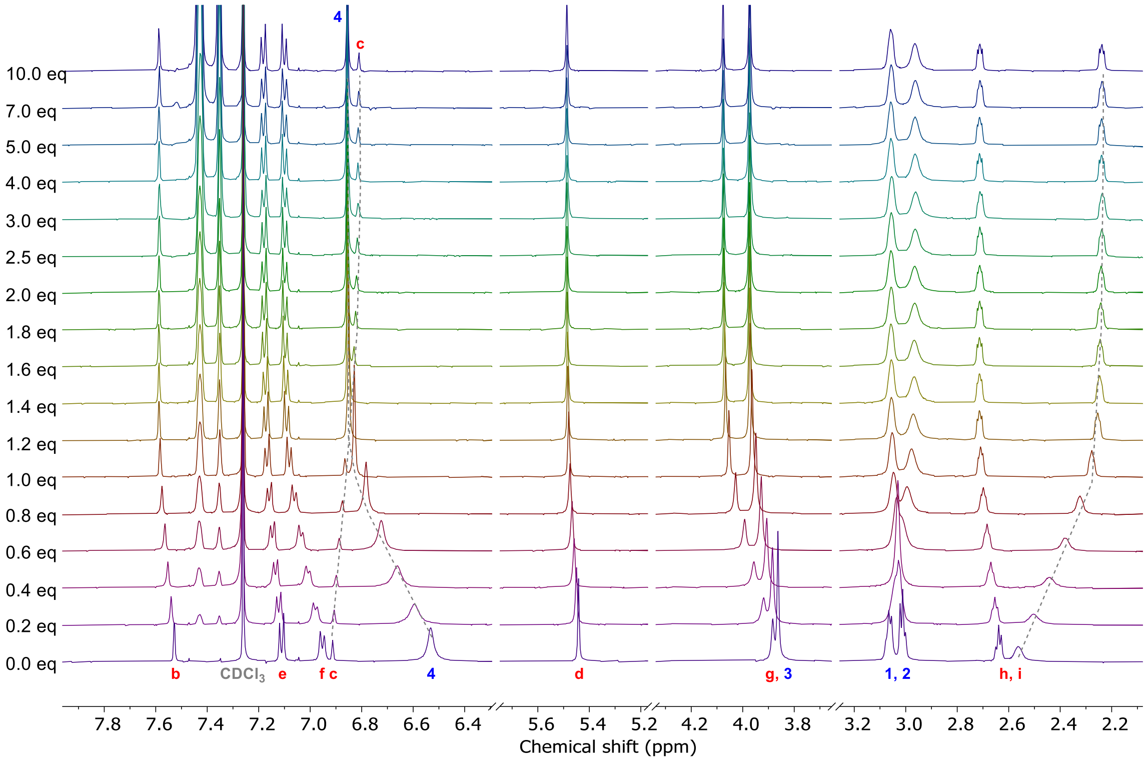

*Figure S7. Truncated ^{1}H NMR titration spectra of [2]catenane **4** upon progressive addition of 10 equivalents $NaBAr^F$ (500 MHz, 298 K, 1:1 $CDCl_3/CD_3CN$, [Receptor] = 1.0 mM).*

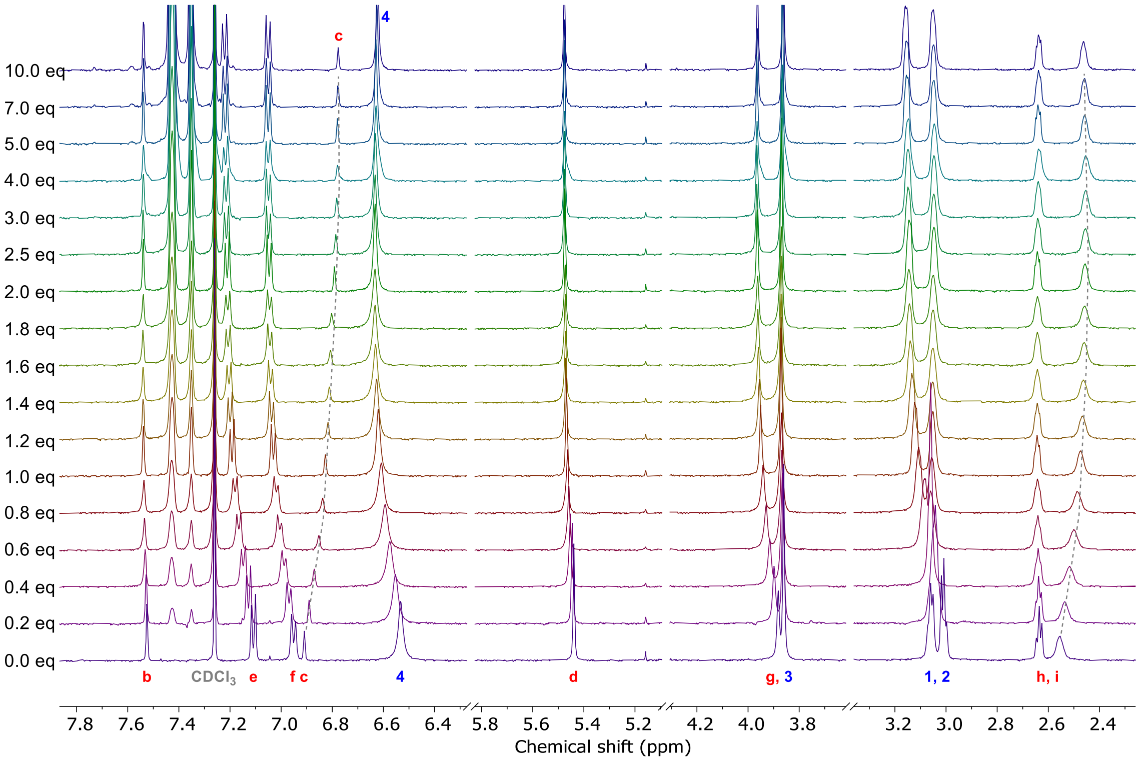

*Figure S8. Truncated ^{1}H NMR titration spectra of [2]catenane **4** upon progressive addition of 10 equivalents KBAr^{F} (500 MHz, 298 K, 1:1 $CDCl_3$/CD_3CN, [Receptor] = 1.0 mM).*

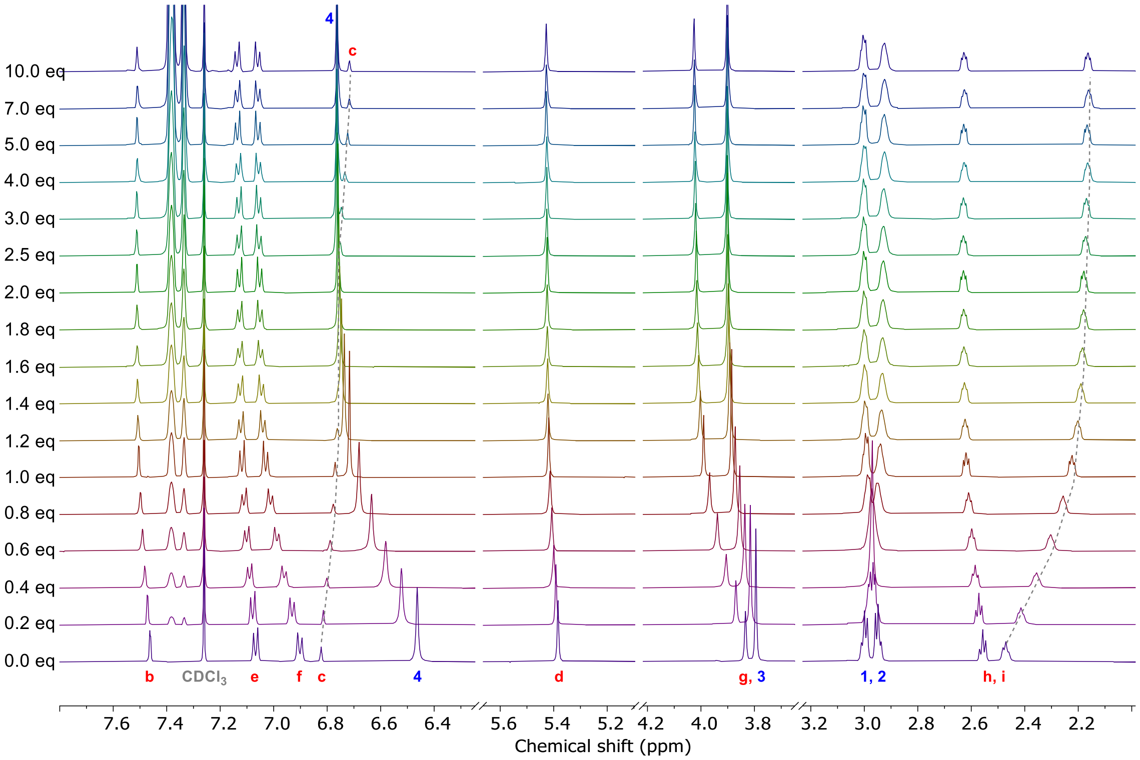

*Figure S9. Truncated ^{1}H NMR titration spectra of [2]catenane **4** upon progressive addition of 10 equivalents NaBAr^{F} (500 MHz, 298 K, 1:3 $CDCl_3$/CD_3CN, [Receptor] = 1.0 mM).*

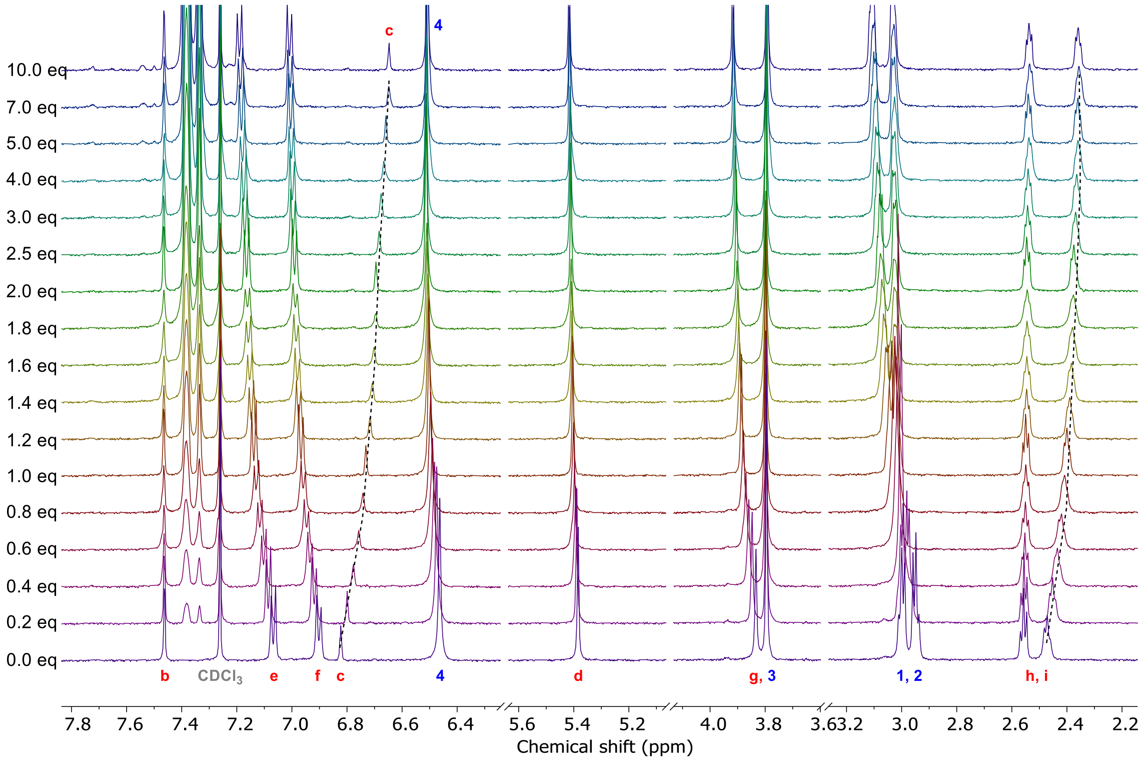

*Figure S10. Truncated ^{1}H NMR titration spectra of [2]catenane **4** upon progressive addition of 10 equivalents KBAr[F] (500 MHz, 298 K, 1:3 $CDCl_3/CD_3CN$, [Receptor] = 1.0 mM).*

Anion titrations

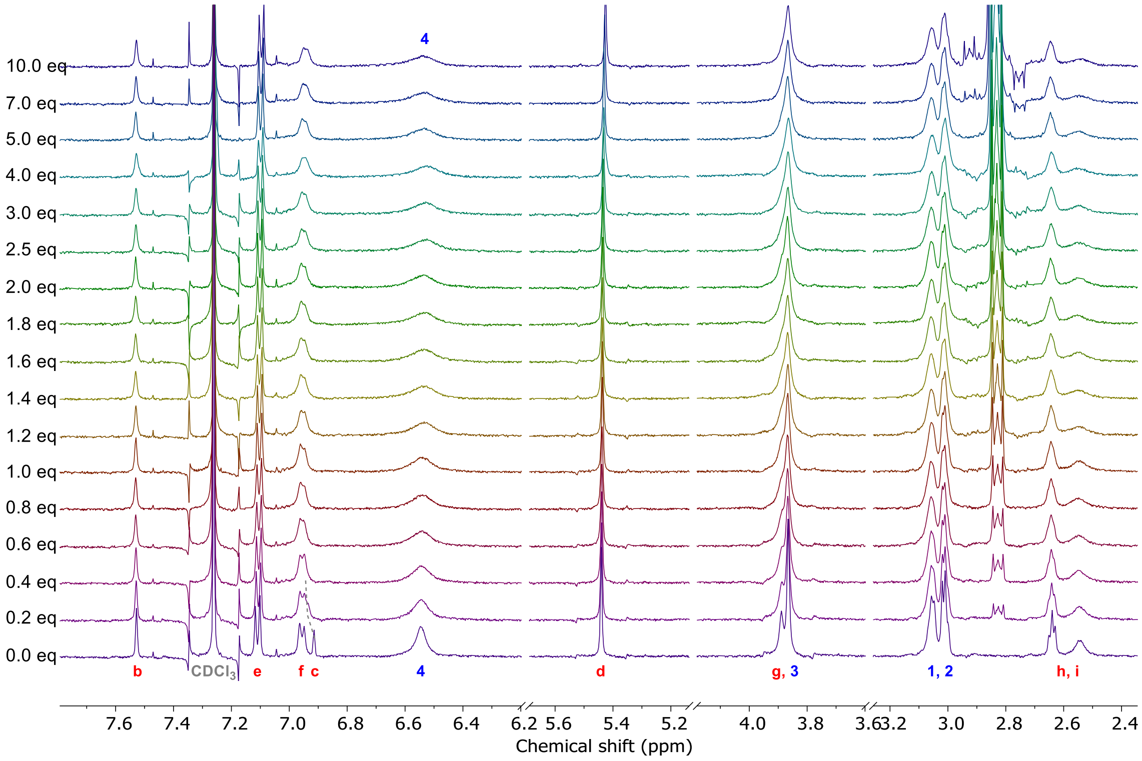

*Figure S11. Truncated ^{1}H NMR titration spectra of [2]catenane **4** upon progressive addition of 10 equivalents TBACl (500 MHz, 298 K, 1:1 $CDCl_3/CD_3CN$, [Receptor] = 1.0 mM).*

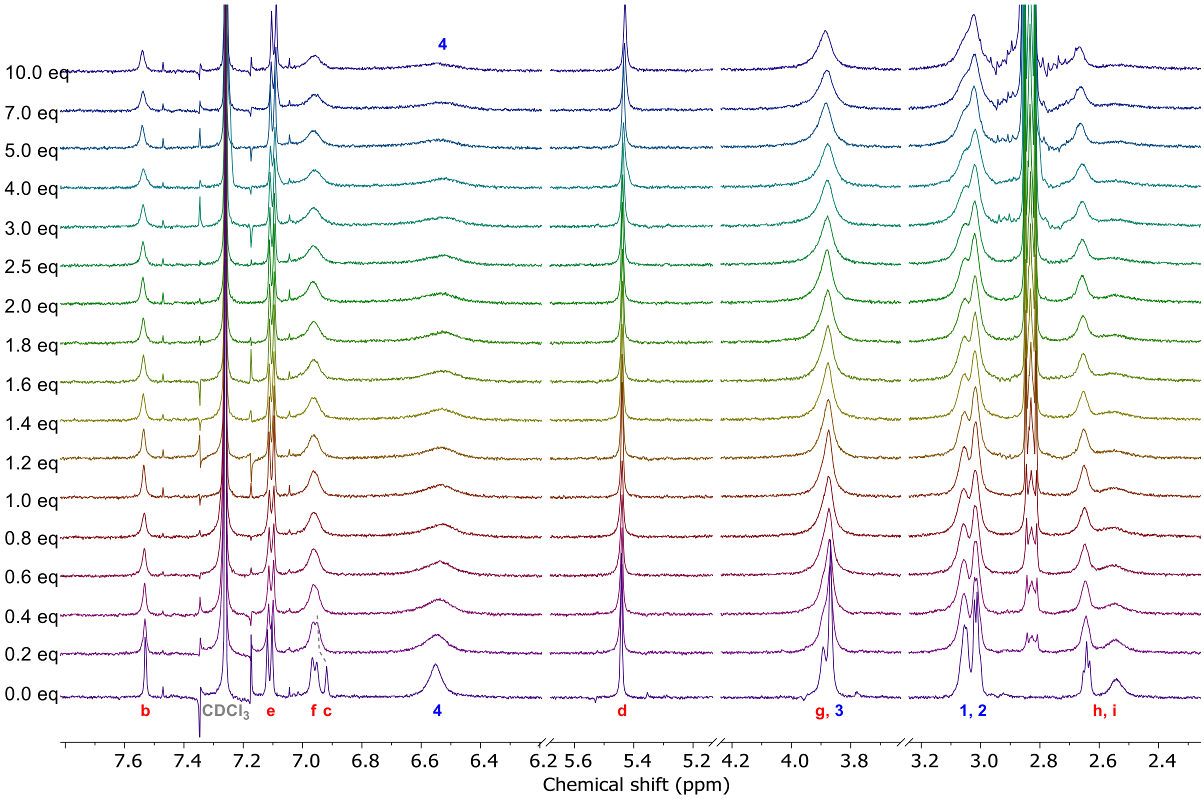

*Figure S12. Truncated ^{1}H NMR titration spectra of [2]catenane **4** upon progressive addition of 10 equivalents TBABr (500 MHz, 298 K, 1:1 $CDCl_3$/CD_3CN, [Receptor] = 1.0 mM).*

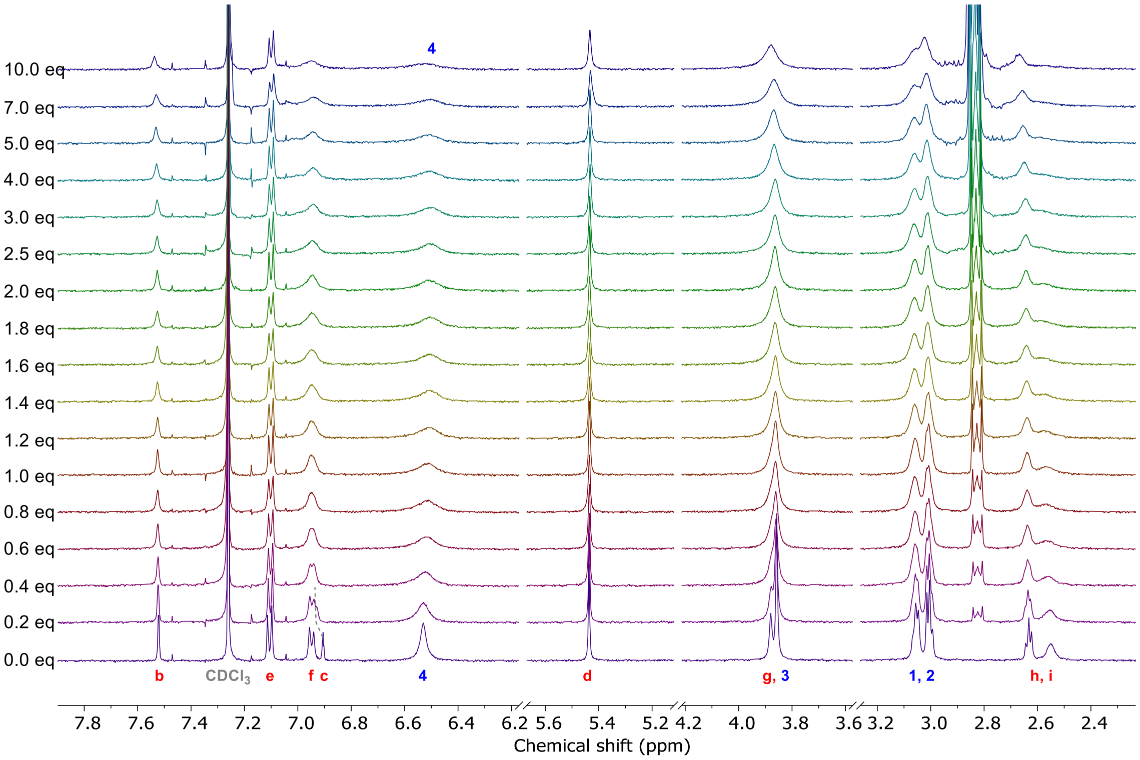

*Figure S13. Truncated ^{1}H NMR titration spectra of [2]catenane **4** upon progressive addition of 10 equivalents TBAI (500 MHz, 298 K, 1:1 $CDCl_3$/CD_3CN, [Receptor] = 1.0 mM).*

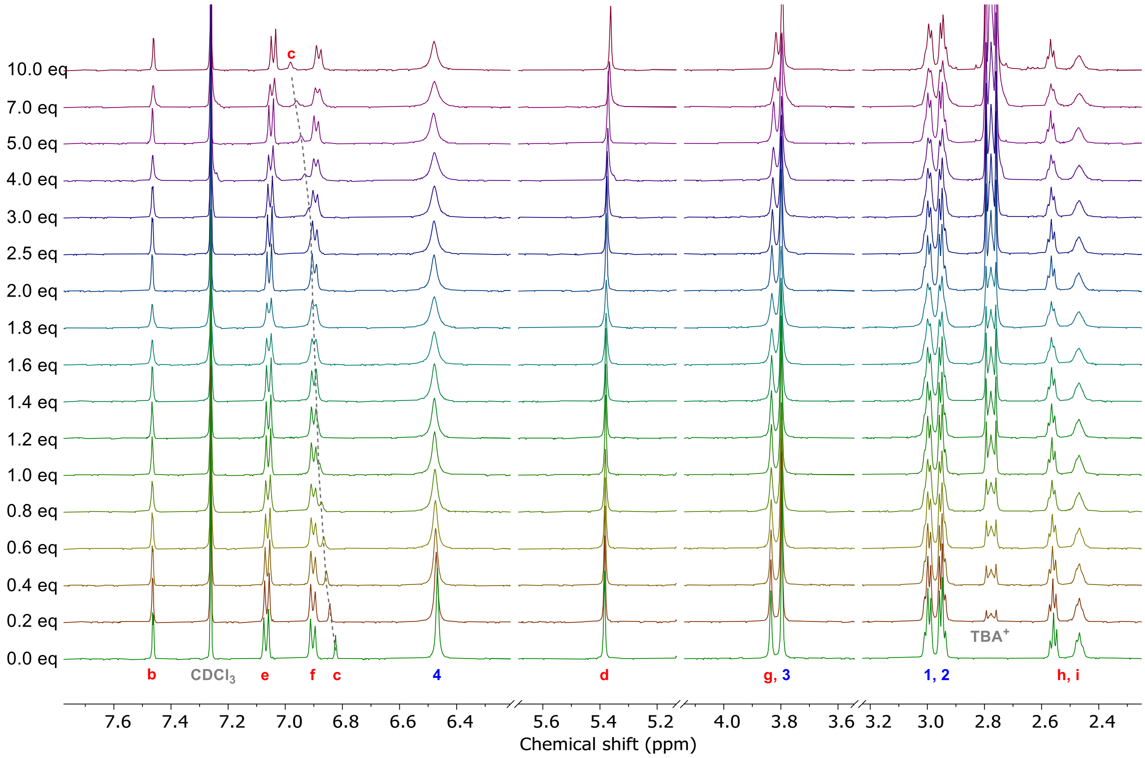

*Figure S14. Truncated ^{1}H NMR titration spectra of [2]catenane **4** upon progressive addition of 10 equivalents TBACl (500 MHz, 298 K, 1:3 $CDCl_3$/CD_3CN, [Receptor] = 1.0 mM).*

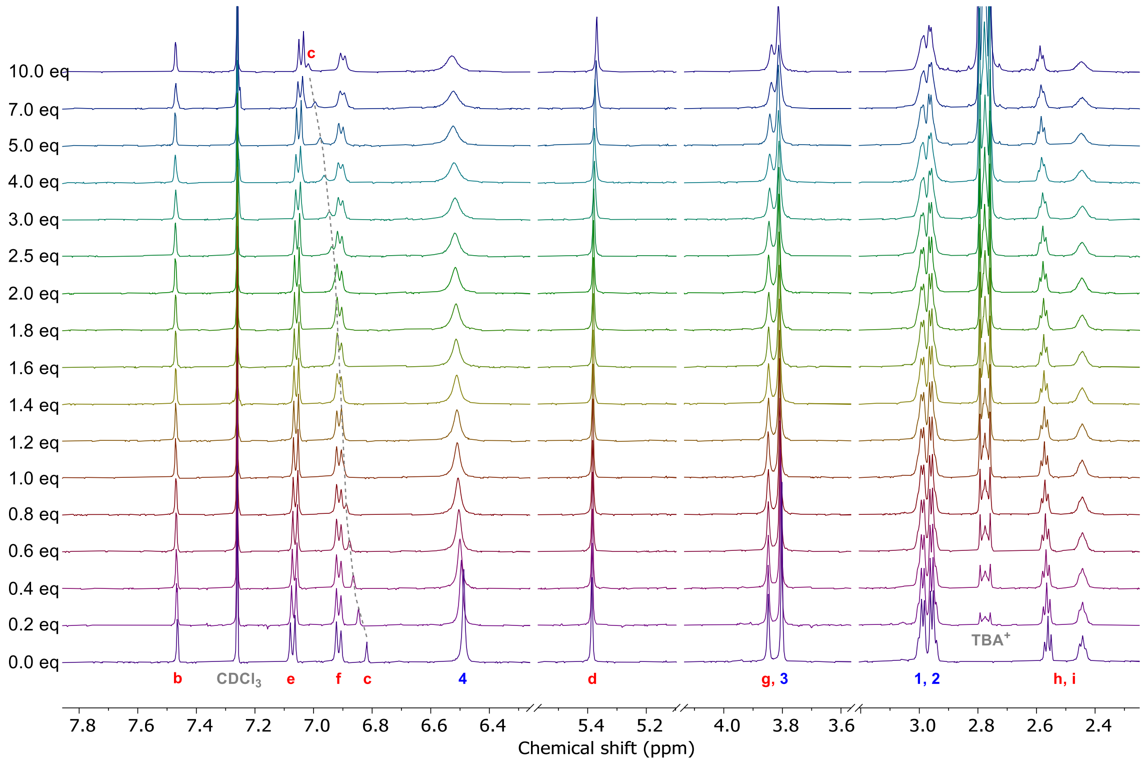

*Figure S15. Truncated ^{1}H NMR titration spectra of [2]catenane **4** upon progressive addition of 10 equivalents TBABr (500 MHz, 298 K, 1:3 $CDCl_3$/CD_3CN, [Receptor] = 1.0 mM).*

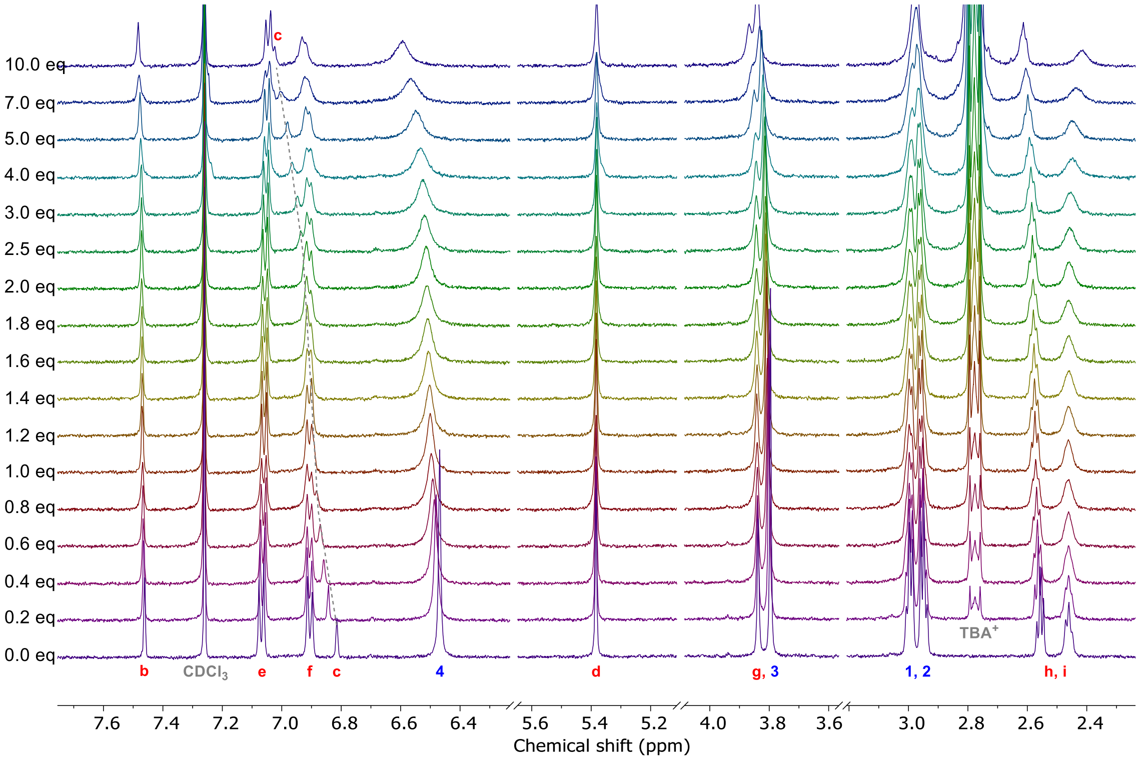

*Figure S16. Truncated ^{1}H NMR titration spectra of [2]catenane **4** upon progressive addition of 10 equivalents TBAI (500 MHz, 298 K, 1:3 $CDCl_3/CD_3CN$, [Receptor] = 1.0 mM).*

Ion-pair titrations

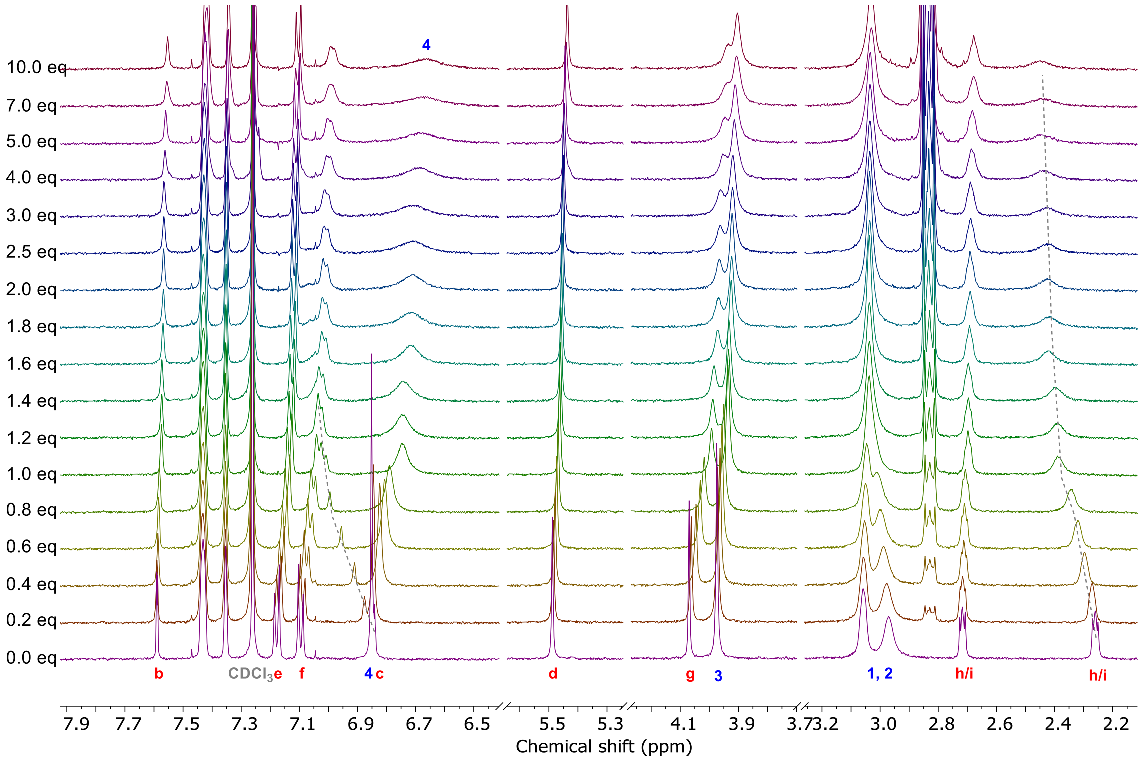

*Figure S17. Truncated ^{1}H NMR titration spectra of [2]catenane **4** upon progressive addition of 10 equivalents TBACl in the presence of 1 equivalent $NaBAr_4^F$ (500 MHz, 298 K, 1:1 $CDCl_3/CD_3CN$, [Receptor] = [$NaBAr_4^F$] = 1.0 mM).*

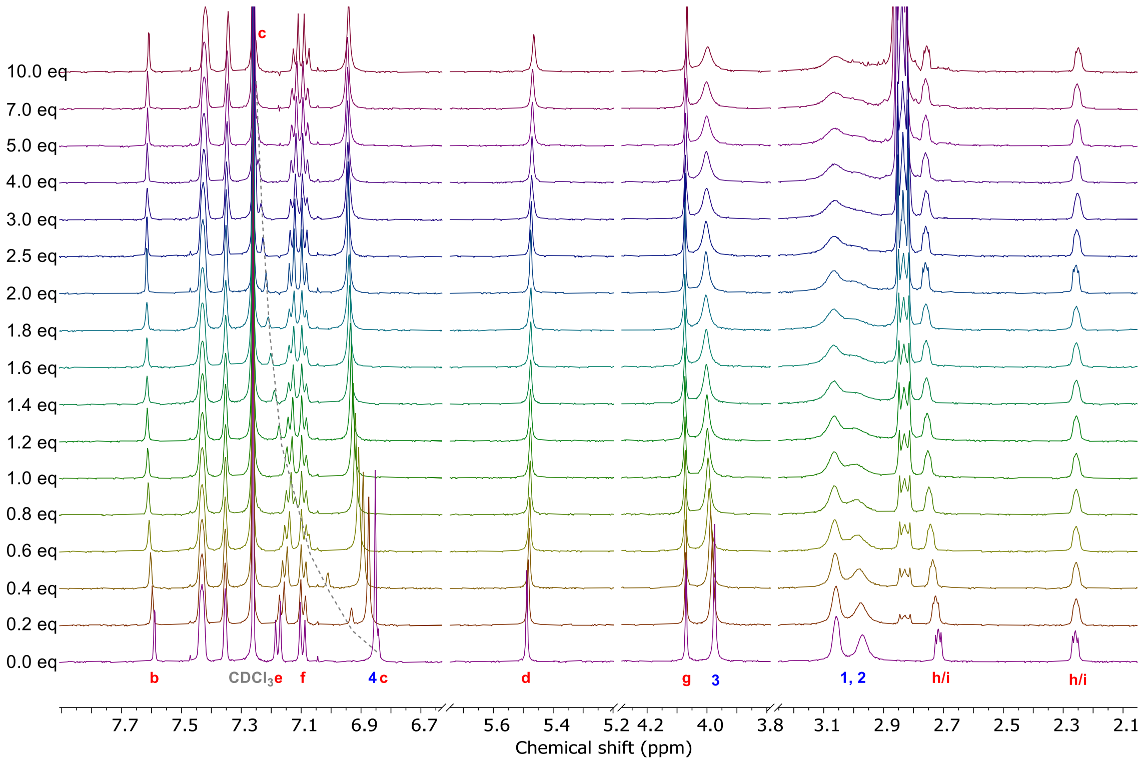

*Figure S18. Truncated ^{1}H NMR titration spectra of [2]catenane **4** upon progressive addition of 10 equivalents TBABr in the presence of 1 equivalent $NaBAr_4^F$ (500 MHz, 298 K, 1:1 $CDCl_3/CD_3CN$, [Receptor] = [$NaBAr_4^F$] = 1.0 mM).*

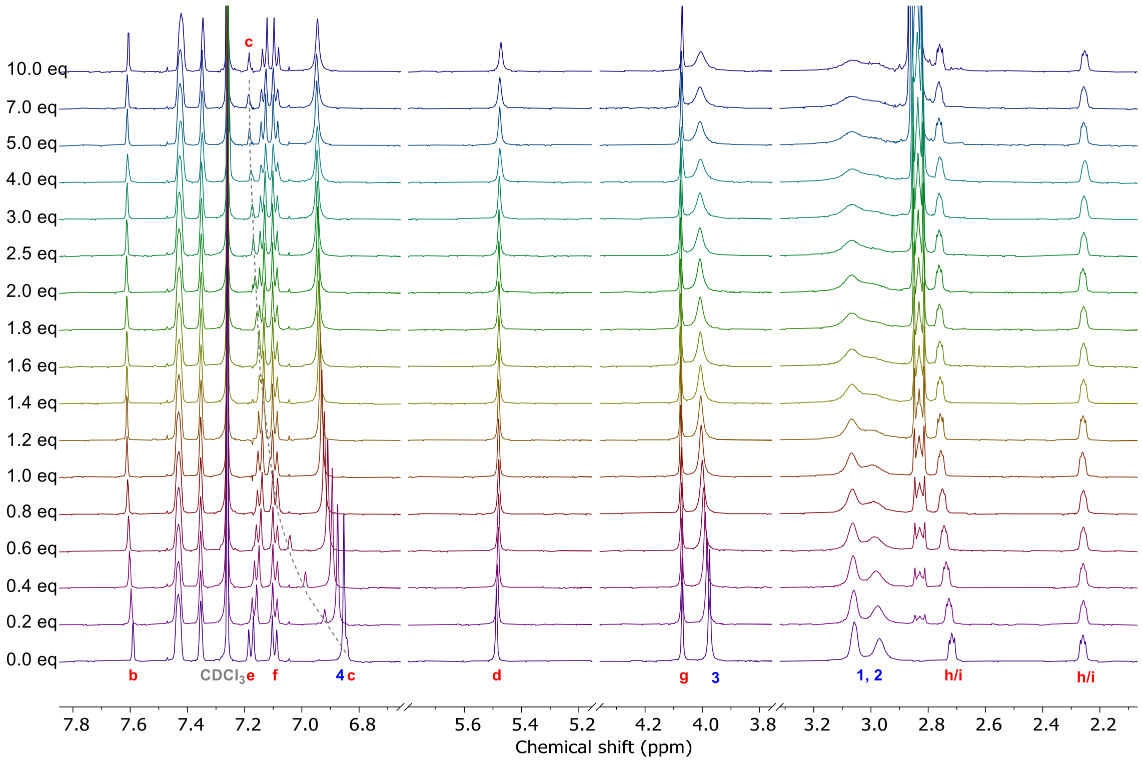

*Figure S19. Truncated ^{1}H NMR titration spectra of [2]catenane **4** upon progressive addition of 10 equivalents TBAI in the presence of 1 equivalent $NaBAr_4^F$ (500 MHz, 298 K, 1:1 $CDCl_3/CD_3CN$, [Receptor] = [$NaBAr_4^F$] = 1.0 mM).*

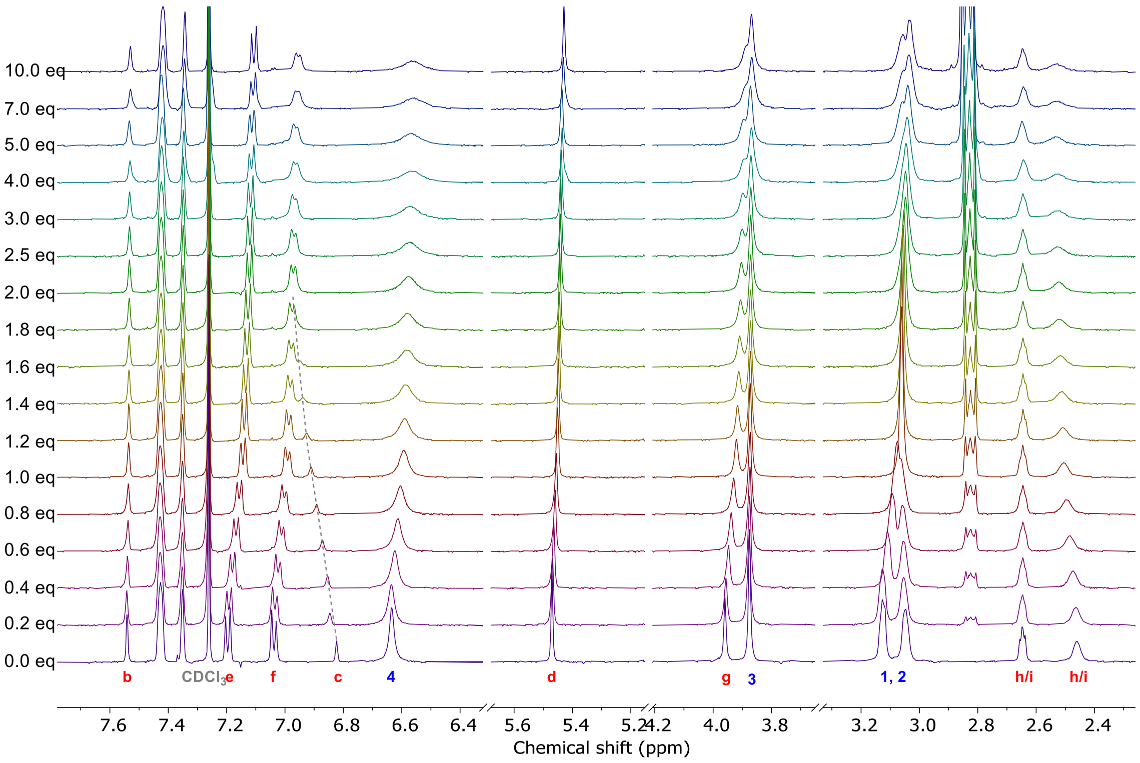

*Figure S20. Truncated ^{1}H NMR titration spectra of [2]catenane **4** upon progressive addition of 10 equivalents TBACl in the presence of 1 equivalent $KBAr_4^F$ (500 MHz, 298 K, 1:1 $CDCl_3/CD_3CN$, [Receptor] = [$KBAr_4^F$] = 1.0 mM).*

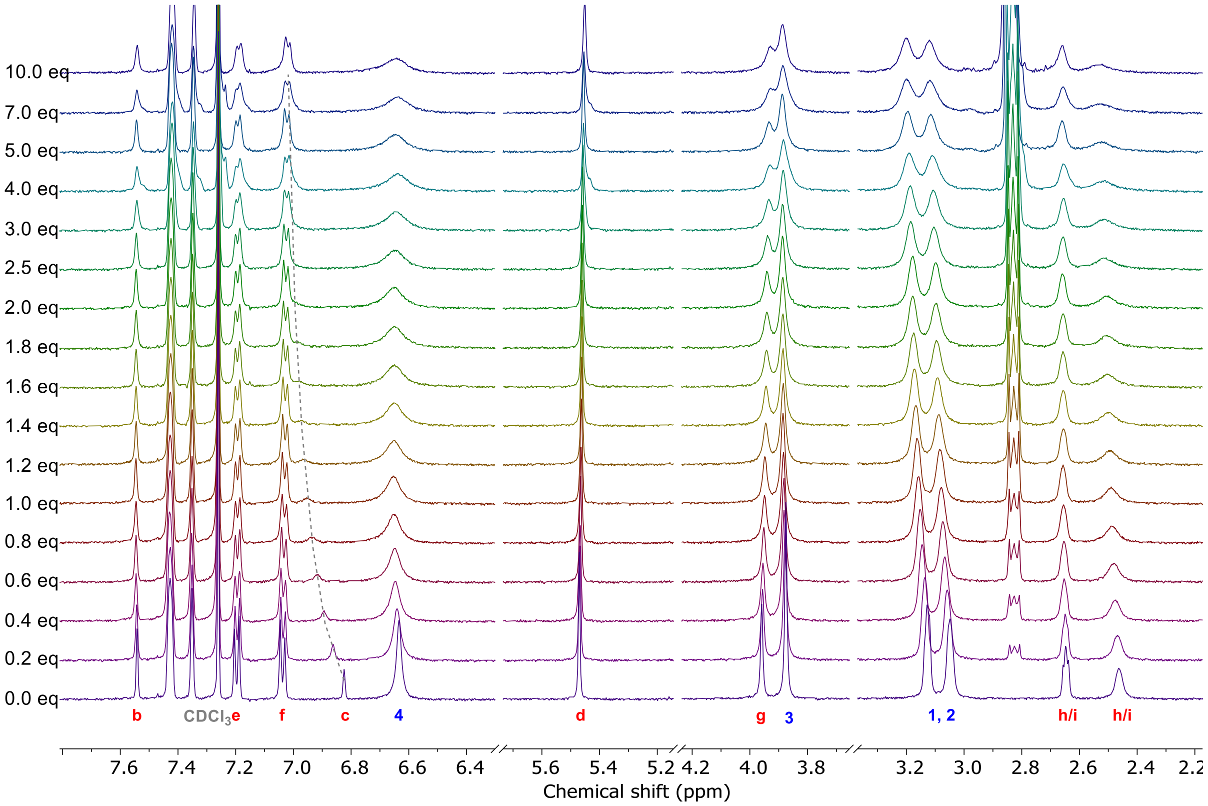

*Figure S21. Truncated ^{1}H NMR titration spectra of [2]catenane **4** upon progressive addition of 10 equivalents TBABr in the presence of 1 equivalent $KBAr_4^F$ (500 MHz, 298 K, 1:1 $CDCl_3/CD_3CN$, [Receptor] = [$KBAr_4^F$] = 1.0 mM).*

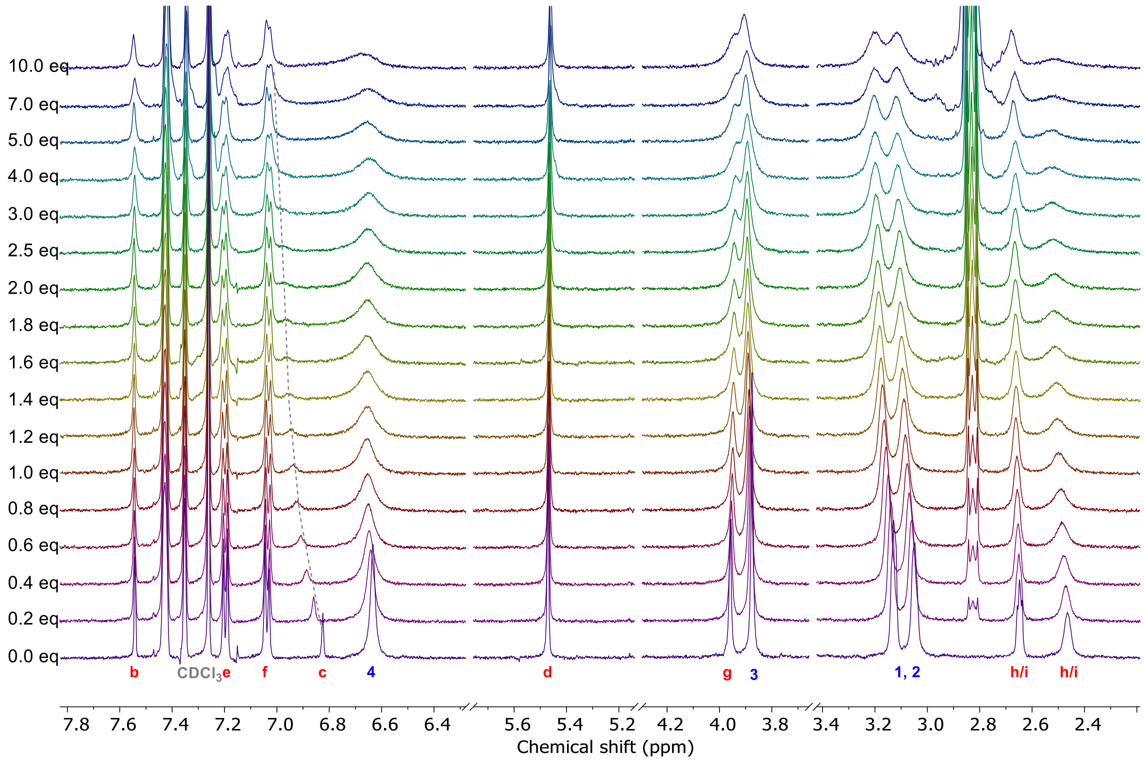

*Figure S22. Truncated ^{1}H NMR titration spectra of [2]catenane **4** upon progressive addition of 10 equivalents TBAI in the presence of 1 equivalent $KBAr_4^F$ (500 MHz, 298 K, 1:1 $CDCl_3/CD_3CN$, [Receptor] = [$KBAr_4^F$] = 1.0 mM).*

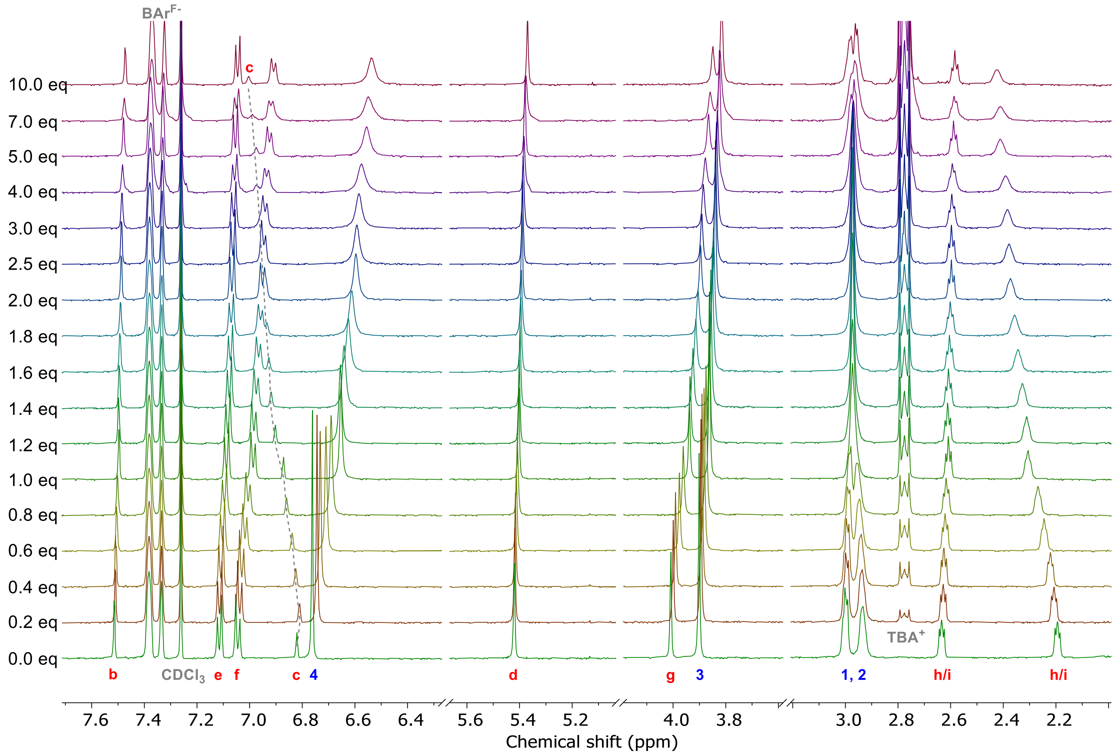

*Figure S23. Truncated ^{1}H NMR titration spectra of [2]catenane **4** upon progressive addition of 10 equivalents TBACl in the presence of 1 equivalent $NaBAr_4^F$ (500 MHz, 298 K, 1:3 $CDCl_3/CD_3CN$, [Receptor] = [$NaBAr_4^F$] = 1.0 mM).*

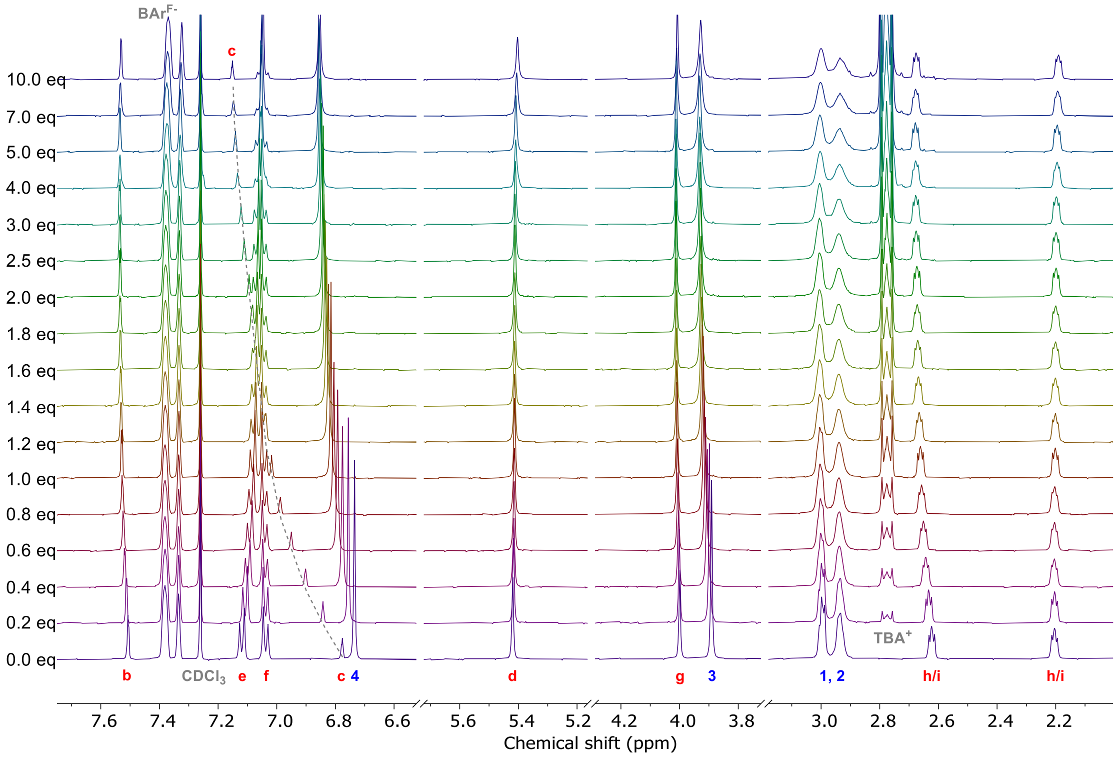

*Figure S24. Truncated ^{1}H NMR titration spectra of [2]catenane **4** upon progressive addition of 10 equivalents TBABr in the presence of 1 equivalent $NaBAr_4^F$ (500 MHz, 298 K, 1:3 $CDCl_3/CD_3CN$, [Receptor] = [$NaBAr_4^F$] = 1.0 mM).*

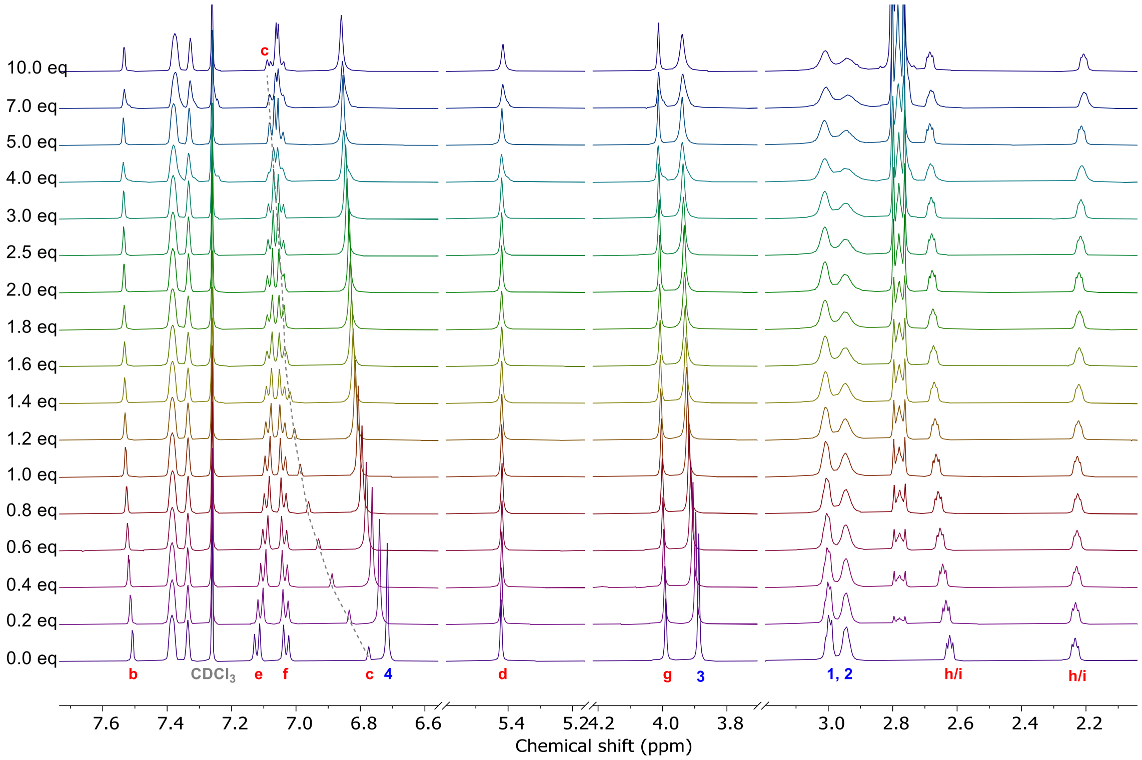

*Figure S25. Truncated ^{1}H NMR titration spectra of [2]catenane **4** upon progressive addition of 10 equivalents TBAI in the presence of 1 equivalent $NaBAr_4^F$ (500 MHz, 298 K, 1:3 $CDCl_3/CD_3CN$, [Receptor] = [$NaBAr_4^F$] = 1.0 mM).*

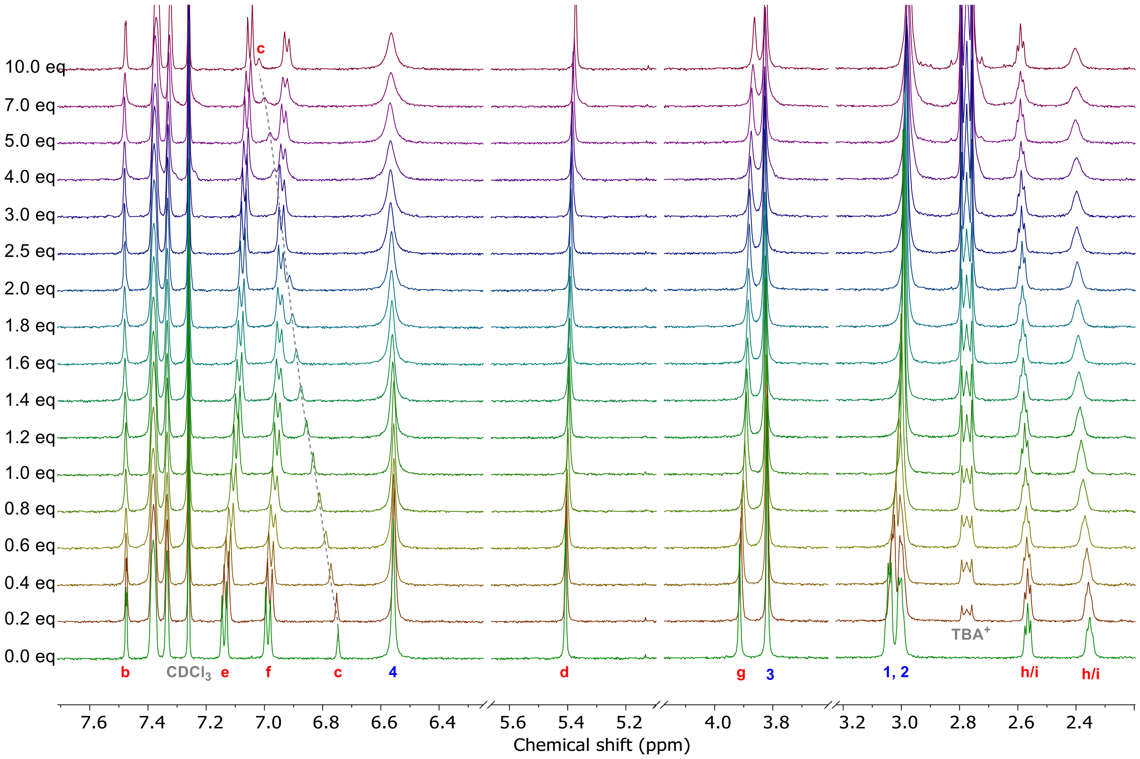

*Figure S26. Truncated ^{1}H NMR titration spectra of [2]catenane **4** upon progressive addition of 10 equivalents TBACl in the presence of 1 equivalent $KBAr_4^F$ (500 MHz, 298 K, 1:3 $CDCl_3/CD_3CN$, [Receptor] = [$KBAr_4^F$] = 1.0 mM).*

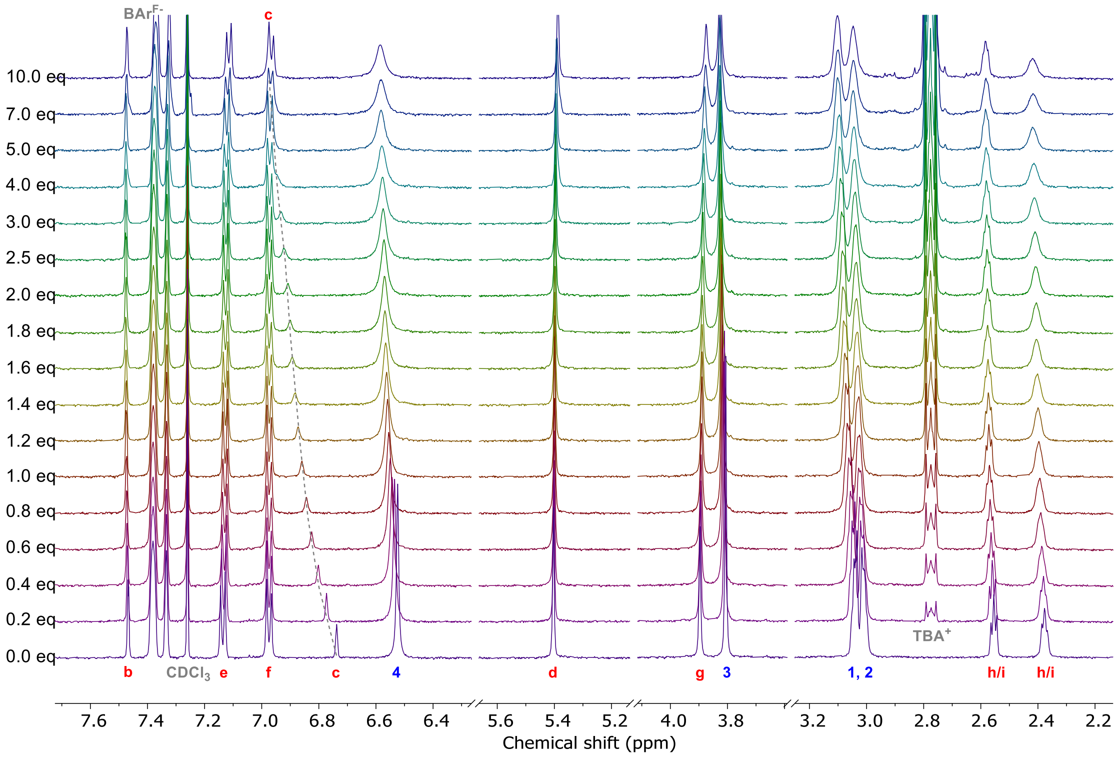

*Figure S27. Truncated ^{1}H NMR titration spectra of [2]catenane **4** upon progressive addition of 10 equivalents TBABr in the presence of 1 equivalent $KBAr_4^F$ (500 MHz, 298 K, 1:3 $CDCl_3/CD_3CN$, [Receptor] = [$KBAr_4^F$] = 1.0 mM).*

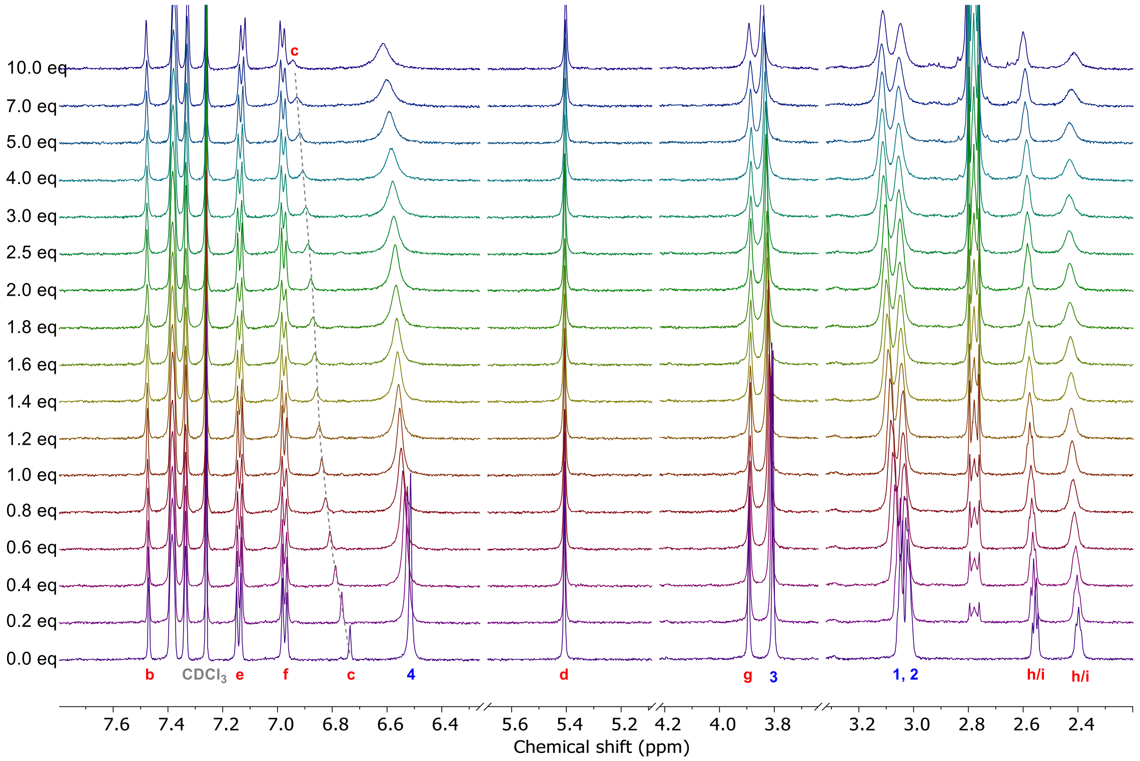

*Figure S28. Truncated ¹H NMR titration spectra of [2]catenane **4** upon progressive addition of 10 equivalents TBAI in the presence of 1 equivalent $KBAr_4^F$ (500 MHz, 298 K, 1:3 $CDCl_3/CD_3CN$, [Receptor] = [$KBAr_4^F$] = 1.0 mM).*

Cation and anion titrations of non-interlocked macrocyclic components

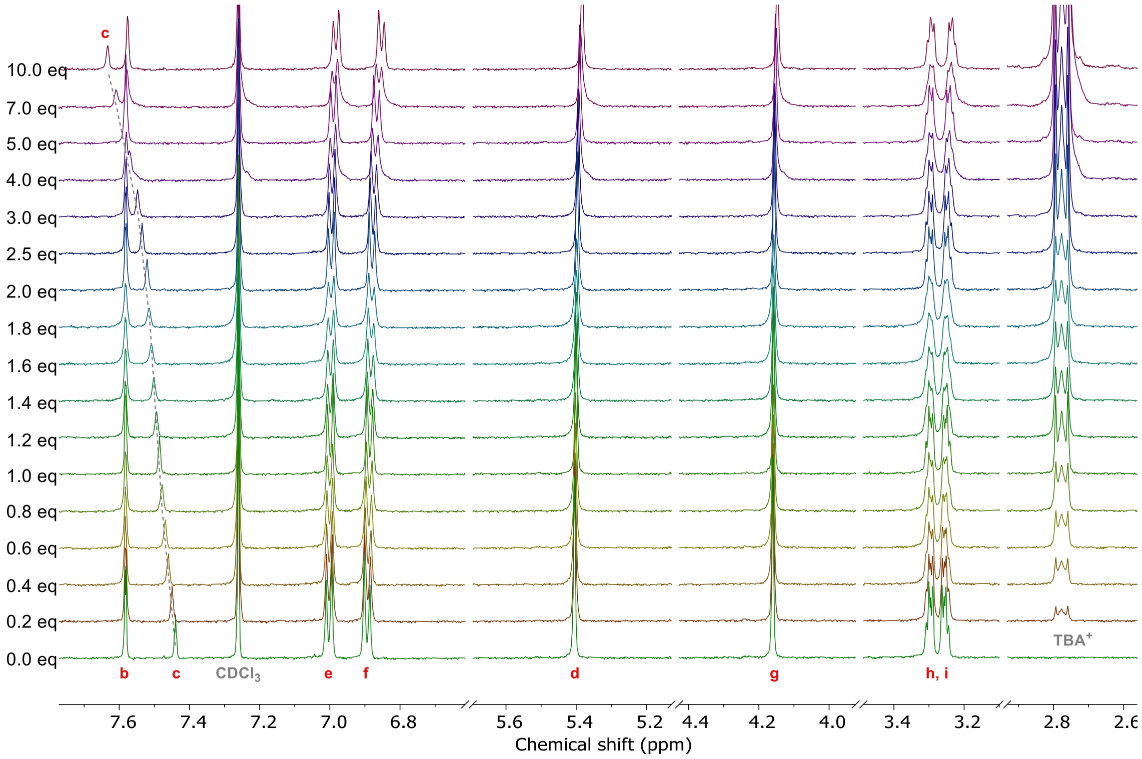

*Figure S29. Truncated ¹H NMR titration spectra of XB macrocycle **5** upon progressive addition of 10 equivalents TBACl (500 MHz, 298 K, 1:3 $CDCl_3/CD_3CN$, [Receptor] = 1.0 mM).*

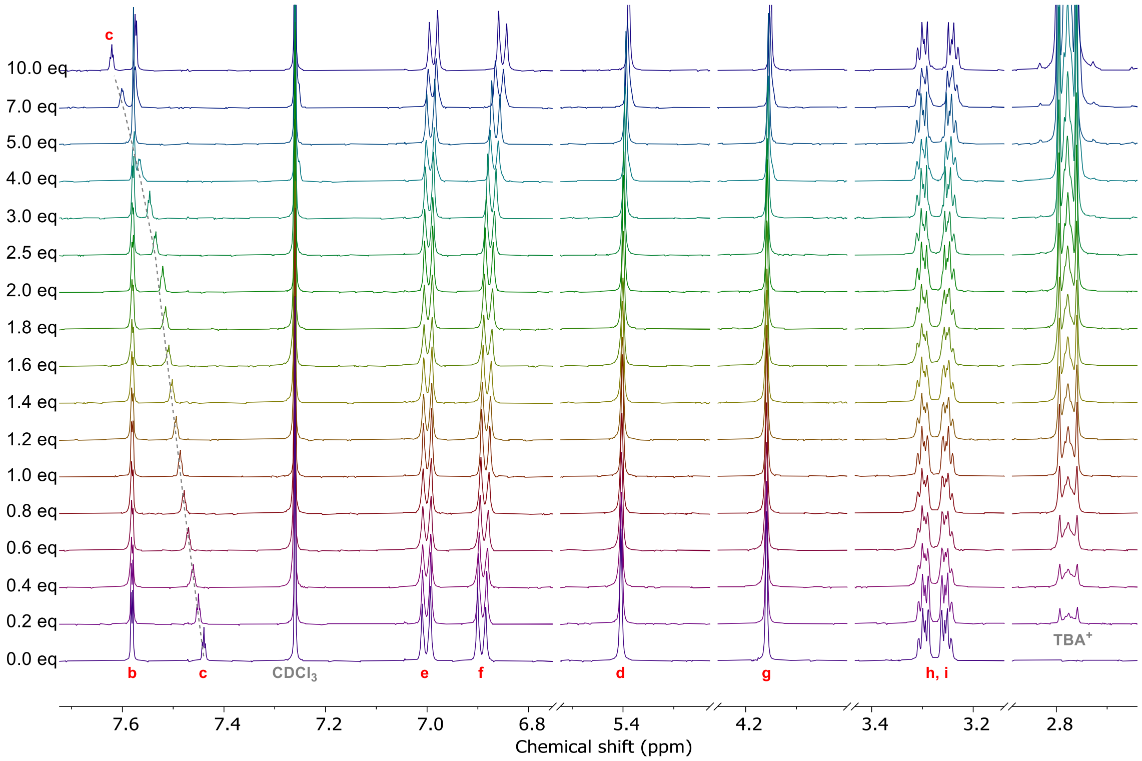

*Figure S30. Truncated ^{1}H NMR titration spectra of XB macrocycle **5** upon progressive addition of 10 equivalents TBABr (500 MHz, 298 K, 1:3 $CDCl_3/CD_3CN$, [Receptor] = 1.0 mM).*

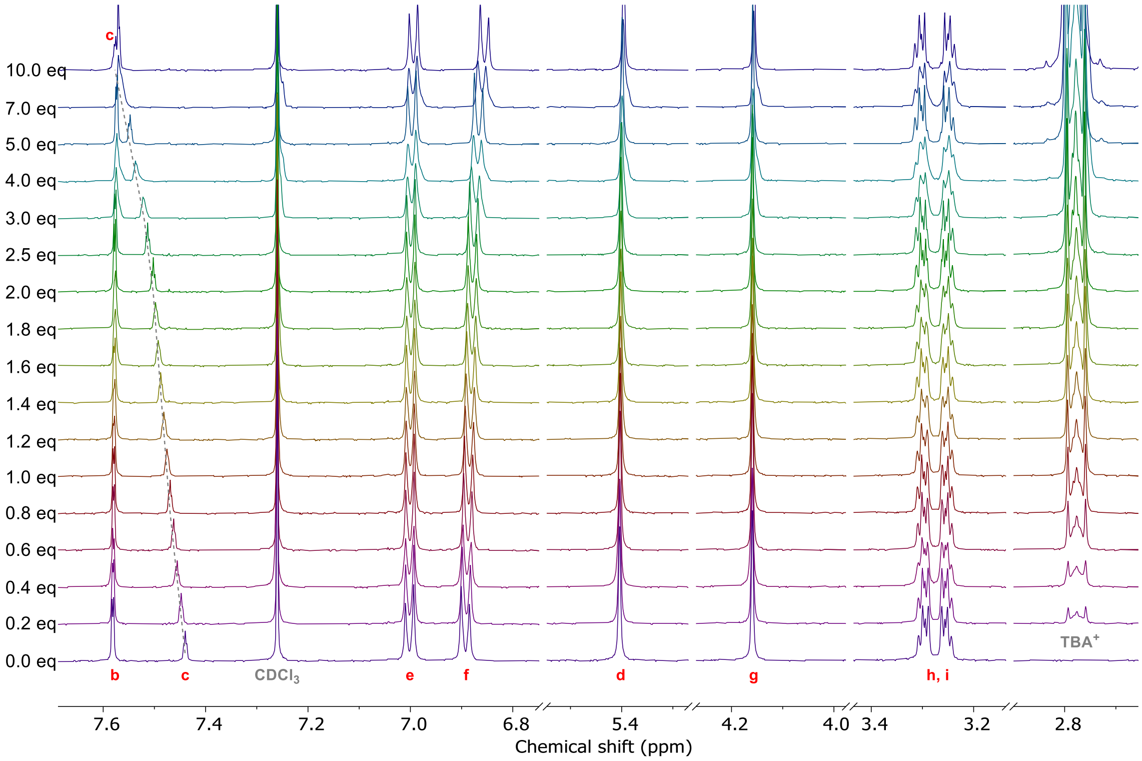

*Figure S31. Truncated ^{1}H NMR titration spectra of XB macrocycle **5** upon progressive addition of 10 equivalents TBAI (500 MHz, 298 K, 1:3 $CDCl_3/CD_3CN$, [Receptor] = 1.0 mM).*

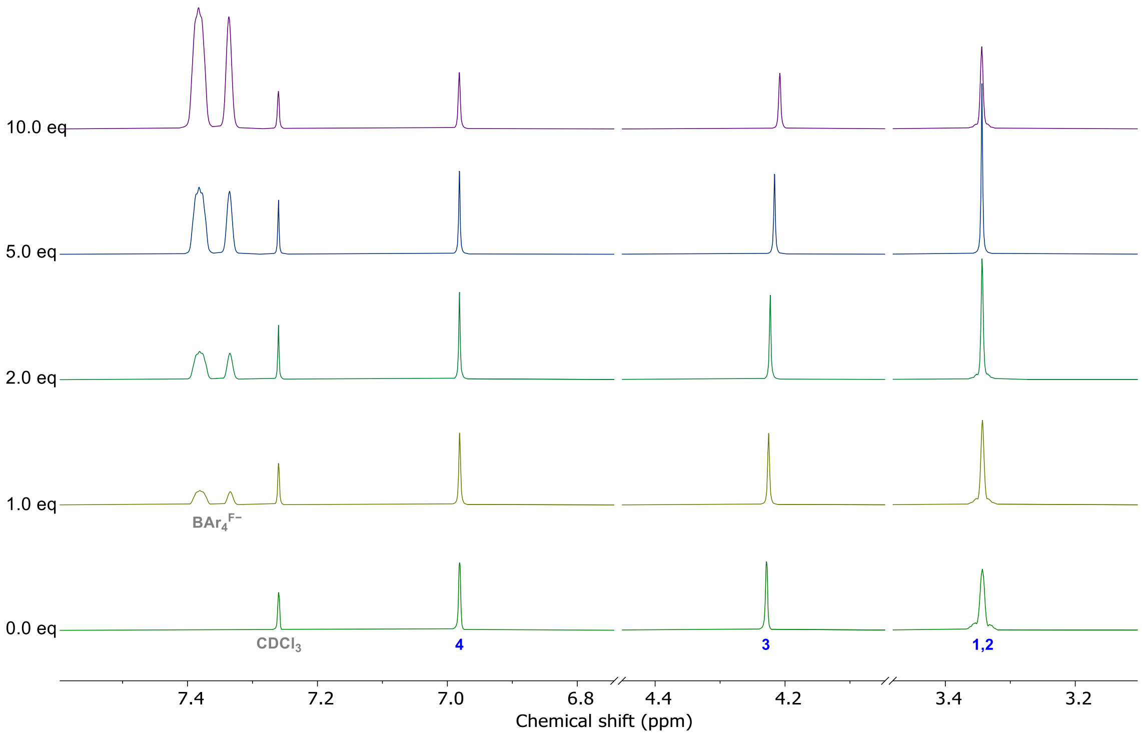

*Figure S32. Truncated ^{1}H NMR titration spectra of macrocycle **1** upon progressive addition of 10 equivalents NaBAr^{F} (500 MHz, 298 K, 1:1 $CDCl_3/CD_3CN$, [Receptor] = 1.0 mM).*

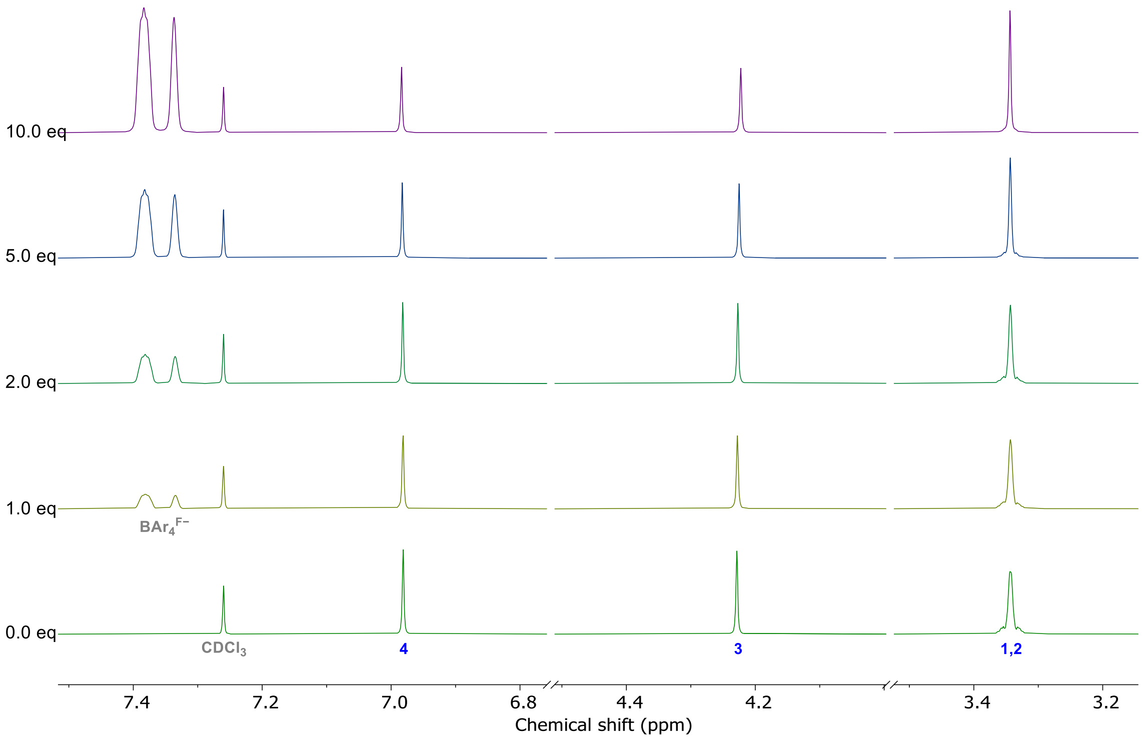

*Figure S33. Truncated ^{1}H NMR titration spectra of macrocycle **1** upon progressive addition of 10 equivalents KBAr^{F} (500 MHz, 298 K, 1:1 $CDCl_3/CD_3CN$, [Receptor] = 1.0 mM).*

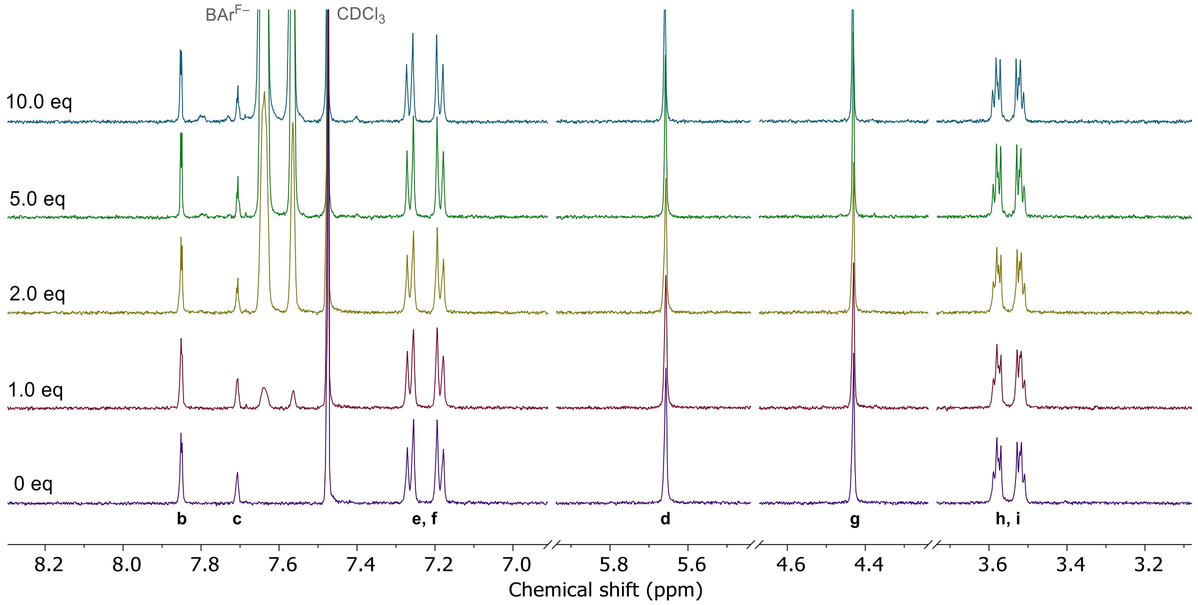

*Figure S34. Truncated ^{1}H NMR titration spectra of macrocycle **5** upon progressive addition of 10 equivalents NaBAr^F (500 MHz, 298 K, 1:1 $CDCl_3$/CD_3CN, [Receptor] = 1.0 mM).*

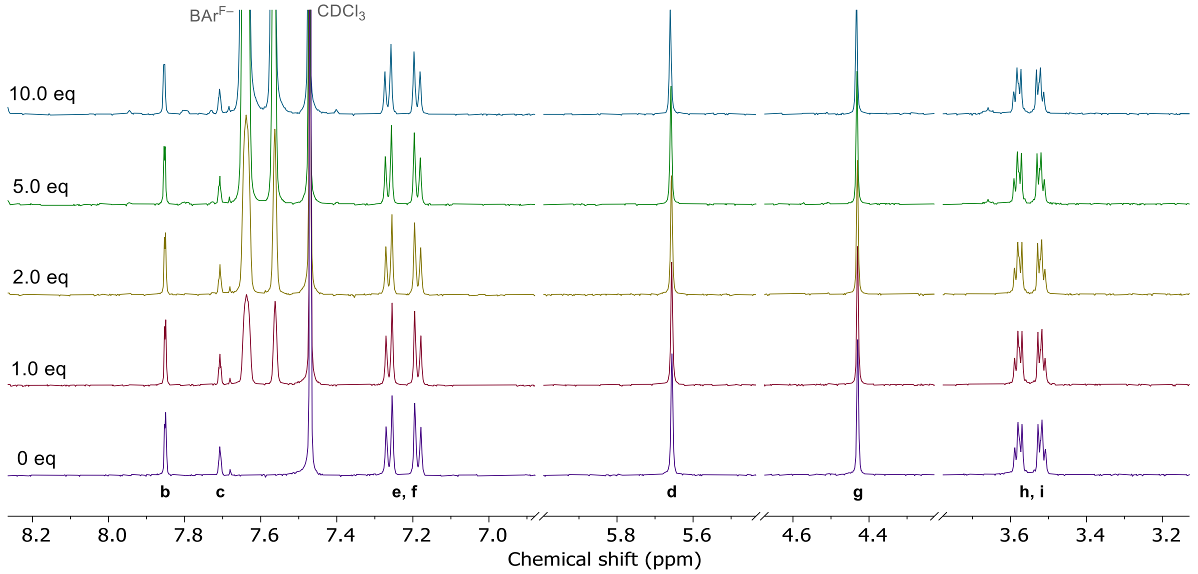

*Figure S35. Truncated ^{1}H NMR titration spectra of macrocycle **5** upon progressive addition of 10 equivalents KBAr^F (500 MHz, 298 K, 1:1 $CDCl_3$/CD_3CN, [Receptor] = 1.0 mM).*

Binding Isotherms

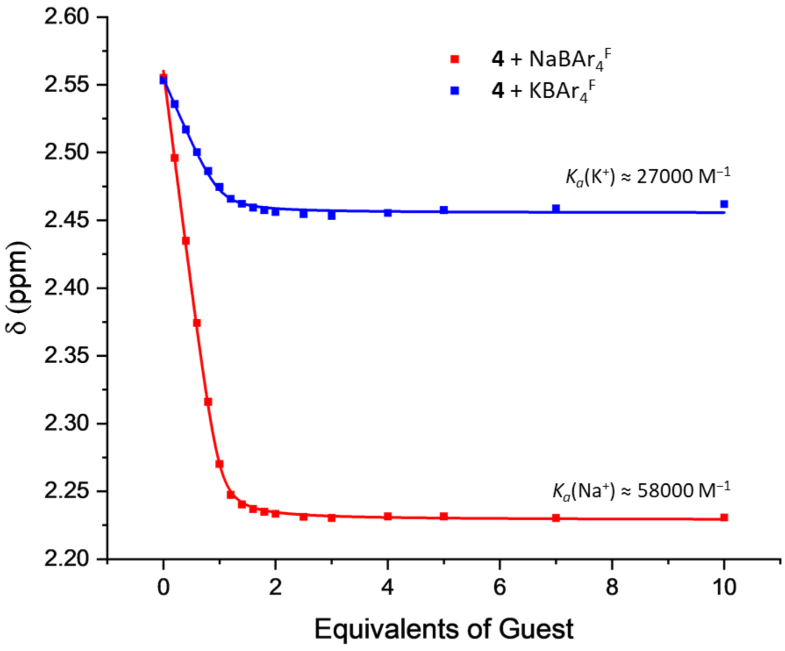

*Figure S36. Binding isotherms of hetero{2]catenane **4**, showing changes in chemical shift of the ethylene glycol protons $H_{h/i}$ with increasing equivalents of $MBAr^F$ salts (M = Na, K). ([Receptor] = 1.0 mM, 500 MHz, 298 K, 1:1 $CDCl_3$:CD_3CN)*

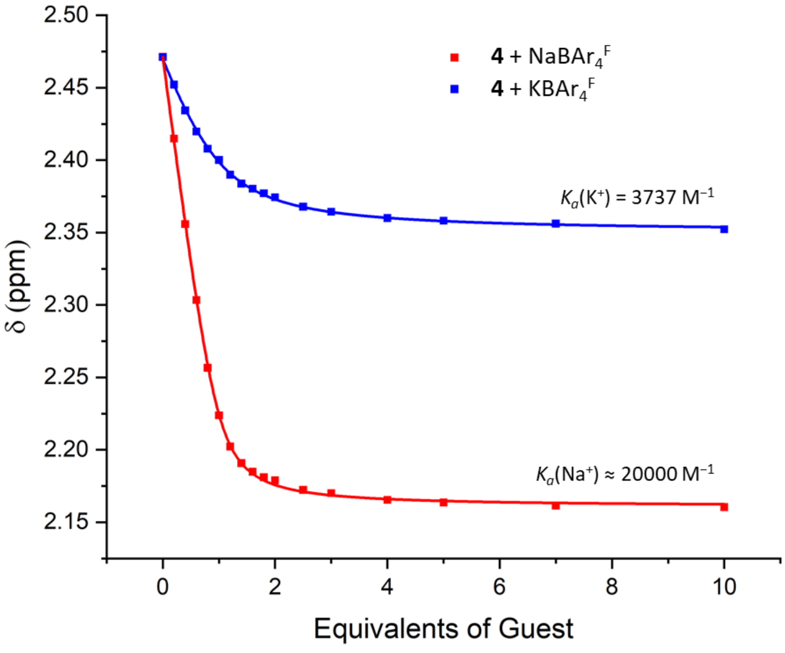

*Figure S37. Binding isotherms of hetero{2]catenane **4**, showing changes in chemical shift of the ethylene glycol protons $H_{h/i}$ with increasing equivalents of $MBAr^F$ salts (M = Na, K). ([Receptor] = 1.0 mM, 500 MHz, 298 K, 1:3 $CDCl_3$:CD_3CN)*

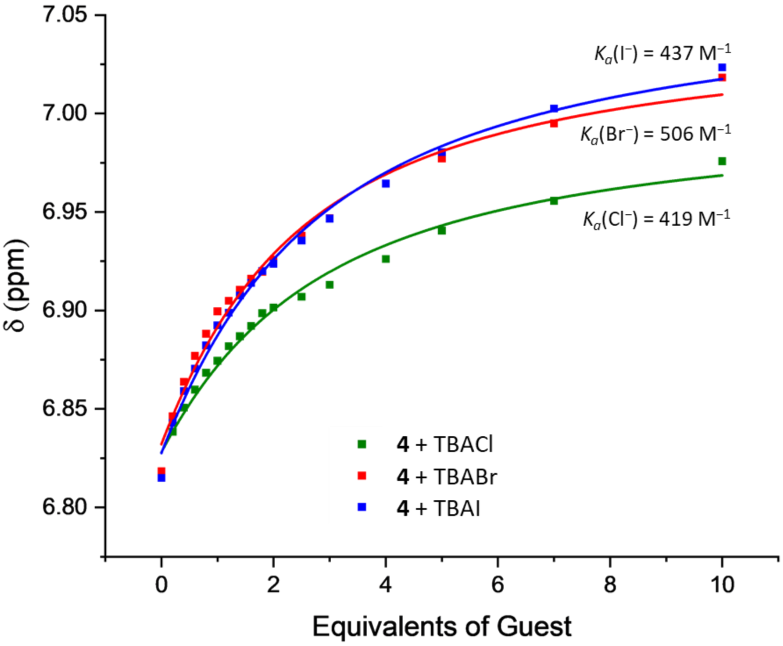

*Figure S38. Binding isotherms of hetero{2]catenane **4**, showing changes in chemical shift of the internal benzene proton H_c with increasing equivalents of TBAX salts (X = Cl, Br, I). ([Receptor] = 1.0 mM, 500 MHz, 298 K, 1:3 $CDCl_3$:CD_3CN)*

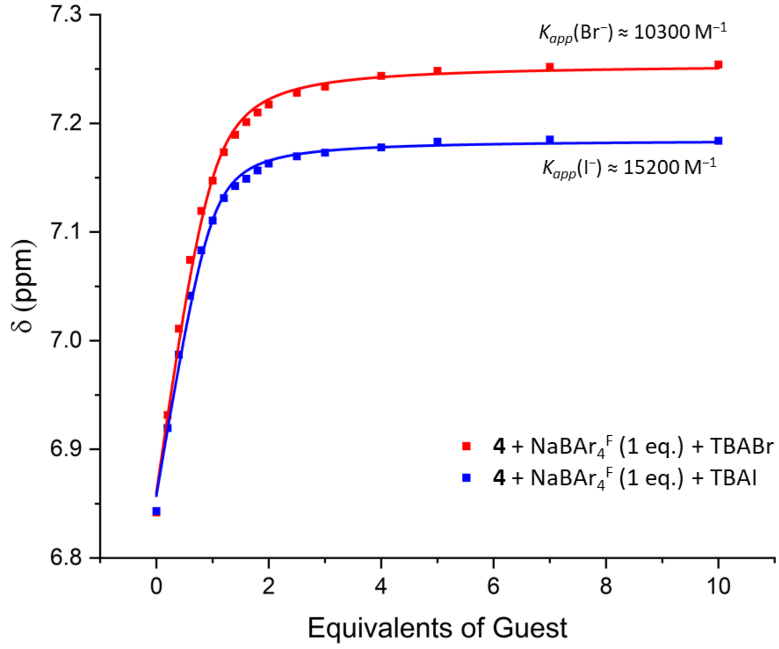

*Figure S39. Binding isotherms of hetero{2]catenane **4**, showing changes in chemical shift of the internal benzene proton H_c with increasing equivalents of TBAX salts (X = Br, I) in the presence of 1 eq. $NaBAr_4^F$. ([Receptor] = 1.0 mM, 500 MHz, 298 K, 1:1 $CDCl_3$:CD_3CN)*

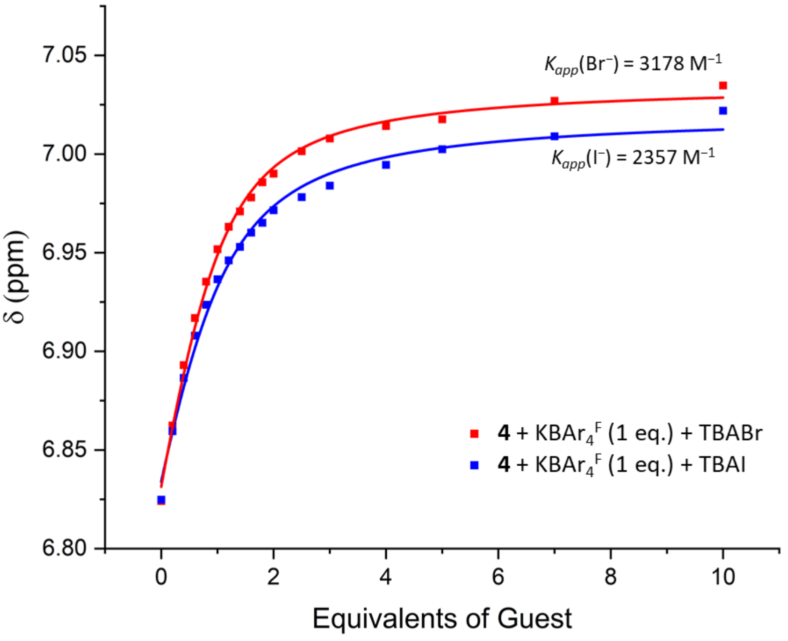

*Figure S40. Binding isotherms of hetero{2]catenane **4**, showing changes in chemical shift of the internal benzene proton H_c with increasing equivalents of TBAX salts (X = Br, I) in the presence of 1 eq. KBAr$_4^F$. ([Receptor] = 1.0 mM, 500 MHz, 298 K, 1:1 $CDCl_3$:CD_3CN)*

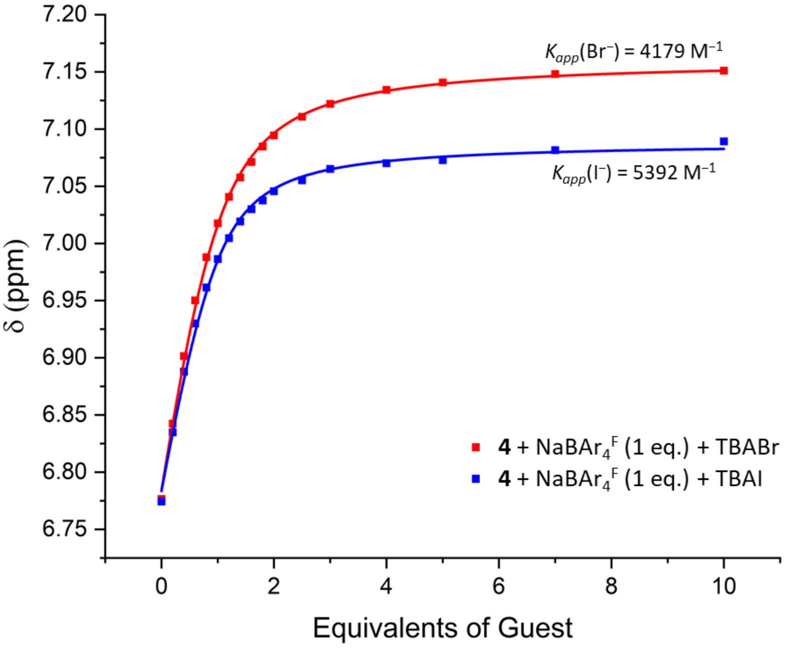

*Figure S41. Binding isotherms of hetero{2]catenane **4**, showing changes in chemical shift of the internal benzene proton H_c with increasing equivalents of TBAX salts (X = Br, I) in the presence of 1 eq. NaBAr$_4^F$. ([Receptor] = 1.0 mM, 500 MHz, 298 K, 1:3 $CDCl_3$:CD_3CN)*

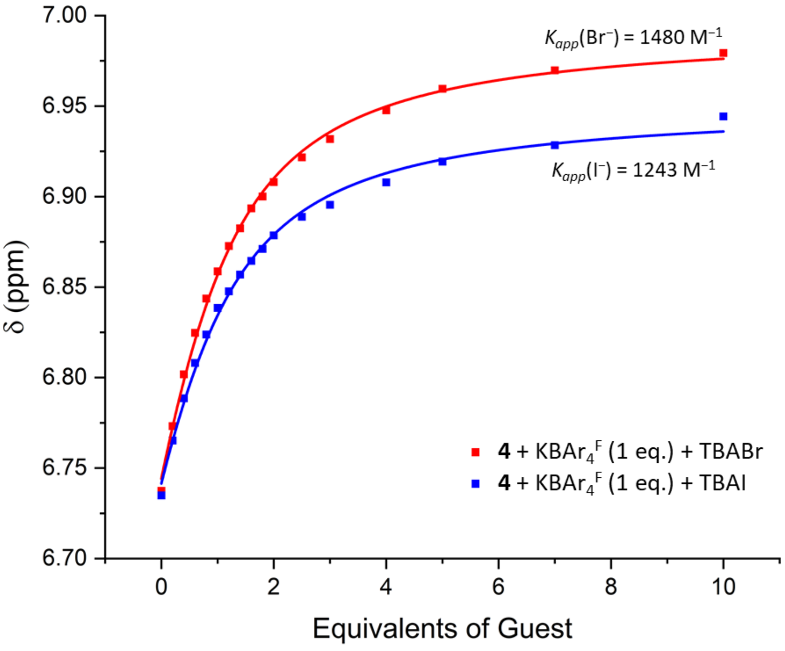

*Figure S42. Binding isotherms of hetero{2]catenane **4**, showing changes in chemical shift of the internal benzene proton H_c with increasing equivalents of TBAX salts (X = Br, I) in the presence of 1 eq. $KBAr_4^F$. ([Receptor] = 1.0 mM, 500 MHz, 298 K, 1:3 $CDCl_3$:CD_3CN)*

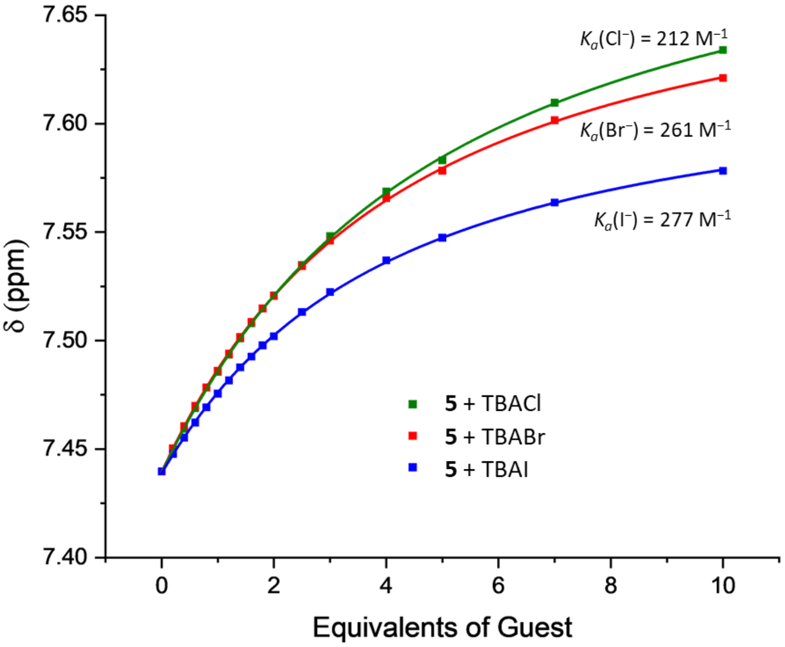

*Figure S43. Binding isotherms of XB macrocycle **5**, showing changes in chemical shift of the internal benzene proton H_c with increasing equivalents of TBAX salts (X = Cl, Br, I). ([Receptor] = 1.0 mM, 500 MHz, 298 K, 1:3 $CDCl_3$:CD_3CN)*

X-ray Crystallographic Studies

General Procedure

Single crystals suitable for X-ray analysis were coated with perfluoropolyether oil, mounted on a 200 µm MiTeGen loop and placed in a cold nitrogen stream (150 K)[5] on an Oxford Diffraction Supernova X-ray diffractometer. Diffraction intensities were measured using monochromated Cu K_{α} diffraction. Data collection, indexing, initial cell refinements, frame integration, final cell refinements and absorption corrections were performed using CrysAlisPro. Crystal structures were solved by SuperFlip[6] or SHELXS[7] and refined using full matrix least-squares on F^2 with CRYSTALS[8] and SHELXL[7] using the Olex2 GUI interface.[9] Hydrogen atoms were included into the model at geometrically calculated positions and refined using a riding model.[10]

More details are included below and in the accompanying CIF which is part of the supplementary data for this manuscript. This data is also provided free of charge by the joint Cambridge Crystallographic Data Centre and Fachinformationszentrum Karlsruhe Access Structures service www.ccdc.cam.ac.uk/structures with deposition number 2348225.

Table S1. Selected crystallographic data for reported structure

**Compound**	**4·NaI**
**Formula**	$C_{59}H_{68}I_3N_6NaO_9$
**Formula Weight**	1396.87
**Temp (K)**	150.15(2)
**Crystal system**	monoclinic
**Space Group**	$P2_1/n$
a **(Å)**	21.3809(4)
b **(Å)**	11.8823(2)
c **(Å)**	26.6796(5)
**α (°)**	90
**β (°)**	100.995(2)
**γ (°)**	90
**Cell Volume ($Å^3$)**	6653.6(2)
Z	4
**Reflections collected (all)**	83698
**Reflections (unique)**	13822
R_{int}	0.0755
R_1 ($I > 2\sigma(I)$)	0.0454
wR_2 (all data)	0.1385

Solid-liquid Extractions

General Procedure

The capability of hetero[2]catenane **4** to extract solid alkali metal salts into organic solvent was investigated through a series of solid-liquid extraction (SLE) experiments. In a typical experiment, 10 mg of a solid alkali metal salt MX (where M = Na, K; X = Br, I) was added to 1.0 mM a solution of the receptor in $CDCl_3$ (700 µL) and the mixture was vigorously sonicated for 20 min. In the competitive solid-liquid extraction experiments, 5 mg of each salt was used. The excess salt was subsequently removed by filtration through a syringe filter. A ^{1}H NMR spectrum of the filtrate was collected using a Bruker AVIII 500 MHz spectrometer at 298 K. Subsequently, 0.125 mL of a 2 mM $CDCl_3$ solution of free catenane **4**, corresponding 0.25 equivalents of the free catenane, was added to each sample and the spectrum re-recorded under the same conditions.

*Pre- and post-extraction ^{1}H NMR spectra of **4***

Treatment of **4** with NaBr (**Figure S44c**) gave rise to distinct peak perturbations consistent with the formation of the **4·NaBr** ion-pair bound complex. In particular, internal benzene proton H_c underwent a dramatic downfield shift relative to the free receptor, indicative of anion binding via XB interactions at the bis(iodotriazole)benzene donor motif. The ethylene glycol protons $H_{h/I}$ split while $H_{1/2}$ broadened, suggesting that the sodium cation is coordinated within the polyether-based interlocked binding cavity. The subsequent addition of 0.25 equivalents of free heterocatenane **4** to this solution resulted in the appearance of a minor set of peaks corresponding to the unbound receptor (**Figure S46b**), thereby confirming the **4·NaBr** ion-pair bound complex exists in slow exchange with the free catenane on the NMR timescale.

In contrast, the post-extraction spectrum of **4** following treatment with KBr featured two distinct set of catenane-derived peaks (**Figure S44b**). The major set corresponds to the free catenane (as shown by the dotted lines connecting **Figures S44a** and **b**), while the minor set exhibits chemical shifts similar to that of the **4·NaBr** ion-pair bound complex (as shown by the dotted lines connecting **Figures S44b** and **c**). Addition of 0.25 equivalents of free heterocatenane **4** to this sample resulted in an increase in the intensity of the major set of peaks relative to the minor set, confirming that these peaks arise from the free receptor. This indicates that the catenane is only capable of partially extracting KBr into solution phase, with the free and bound receptors undergoing slow exchange on the NMR timescale. Integration of the peaks estimates that 43% of the receptor present in solution exists as the ion-pair bound complex. Notably, there are subtle differences in the spectra of **4·NaBr** and **4·KBr** which become apparent upon closer inspection, particularly in the peak shapes of the signals corresponding to H_d, H_g, H_1 and H_2.

In the competitive solid-liquid extraction experiment in which **4** was sonicated with a mixture of NaBr and KBr, the post-extraction spectrum appears virtually identical to that of the **4·NaBr** ion-pair bound complex (**Figure S44d**), suggesting complete extraction of NaBr despite the presence of the competing KBr salt. ESI mass spectrometric analysis of the samples was undertaken in an attempt to verify the identity of the bound cation; however, due to the presence of ubiquitous sodium ions in the solvent system and the strong affinity of **4** for sodium, the major peak observed in all three samples had *m/z* = 1269, corresponding to $[4+Na]^+$.

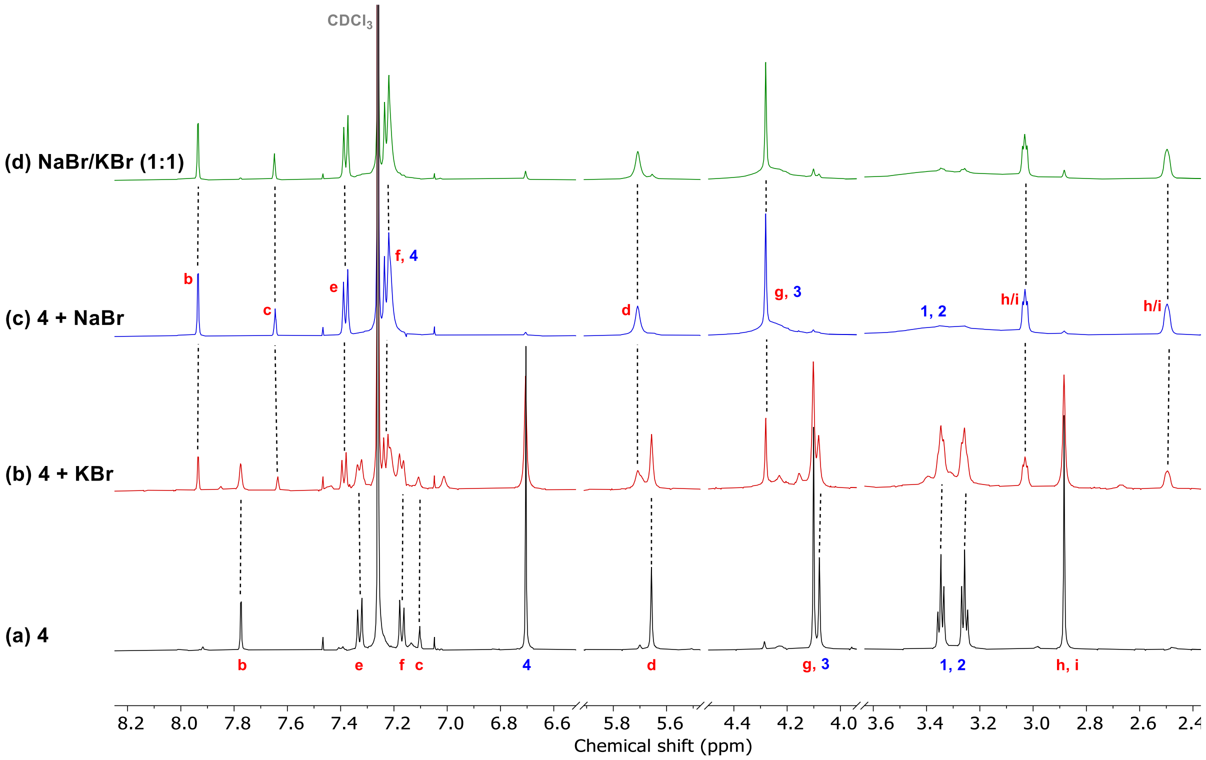

*Figure S44. Solid-liquid extraction studies of **4** with alkali metal bromide salts, showing truncated ^{1}H NMR spectra of: (a) free catenane receptor **4**, and post-extraction spectra of **4** following treatment with (b) 10 mg KBr; (c) 10 mg NaBr; (d) 5 mg NaBr and 5 mg KBr ($CDCl_3$, 298 K, 500 MHz).*

Similar to the NaBr SLE experiment, treatment of **4** with NaI gave rise to a single set of peaks corresponding to the **4·NaI** ion-pair bound complex (**Figure S45c**). A more complex situation was encountered in the solid-liquid extraction experiments of **4** with KI. The post-extraction spectrum after treatment of **4** with KI contains two sets of catenane-derived peaks (**Figure S45b**). In an analogous manner to the KBr experiment, the minor set of peaks, which by integration accounts for 23% of the receptor in solution, exhibits similar chemical shifts to that of the **4·NaI** ion-pair bound complex. However, the major set of peaks (highlighted in **Figure 45b** with asterisks*) exhibits markedly different chemical shifts to that of the free catenane. The subsequent addition of 0.25 equivalents of free catenane **4** to the sample gave rise to a third set of peaks which matches the spectrum of the free receptor. This therefore suggests that the initial two sets of peaks may be due to two different co-conformations of the **4·KI** ion-pair bound complex in slow exchange, perhaps owing to steric clashes between the two large guest ions.

Importantly, treatment of **4** with a mixture of NaI and KI gave rise to a post-extraction spectrum that is identical in appearance to that of the **4·NaI** ion-pair bound complex, once again demonstrating the selective extraction of sodium halides in the presence of the competing potassium halide salt.

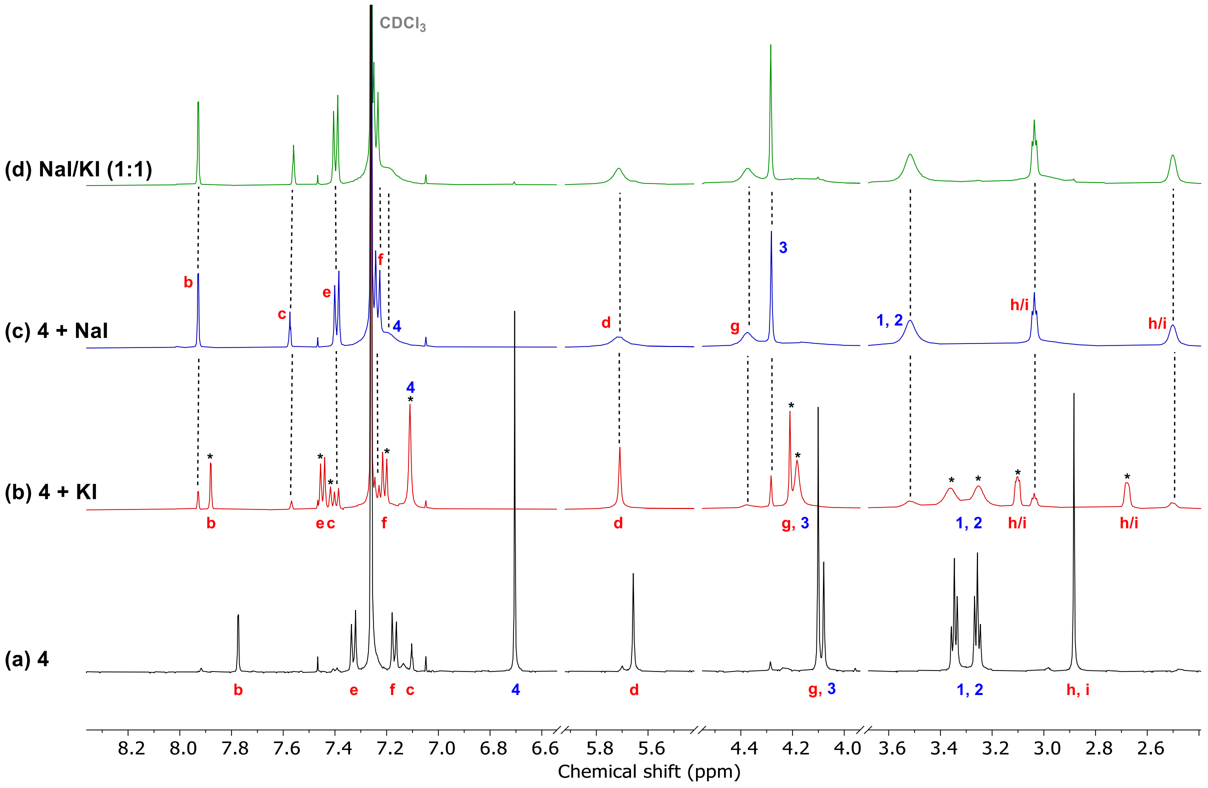

*Figure S45. Solid-liquid extraction studies of **4** with alkali metal iodide salts, showing truncated ^{1}H NMR spectra of: (a) free catenane receptor **4**, and post-extraction spectra of **4** following treatment with (b) 10 mg KI; (c) 10 mg NaI; (d) 5 mg NaI and 5 mg KI ($CDCl_3$, 298 K, 500 MHz).*

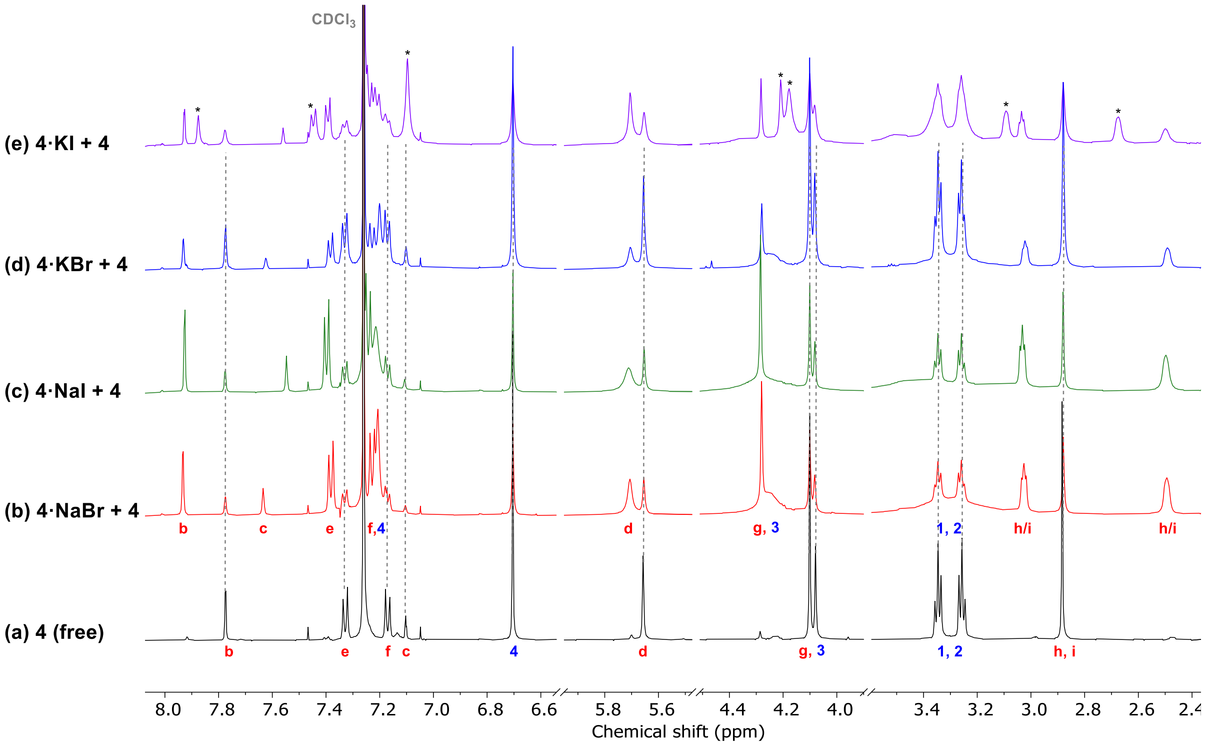

*Figure S46. (a) ^{1}H NMR spectrum of free catenane **4**. (b-e) ^{1}H NMR spectra of **4** after addition of 0.25 equivalents of free catenane to the corresponding post-extraction spectrum with various alkali metal salts. Dotted lines denote the signals arising from the free catenane.*

Analogous SLE experiments conducted on the alkali metal chlorides revealed no significant chemical shift perturbations post-treatment with NaCl and KCl (**Figure S47**), indicating that the ion-pair binding affinity of catenane **4** is insufficient to overcome the high lattice enthalpies of the alkali metal chloride salts. This is consistent with observed salt recombination in the ^{1}H NMR titrations of **4·Na$^+$** and **4·K$^+$** with TBACl in $CDCl_3/CD_3CN$ solvent mixtures.

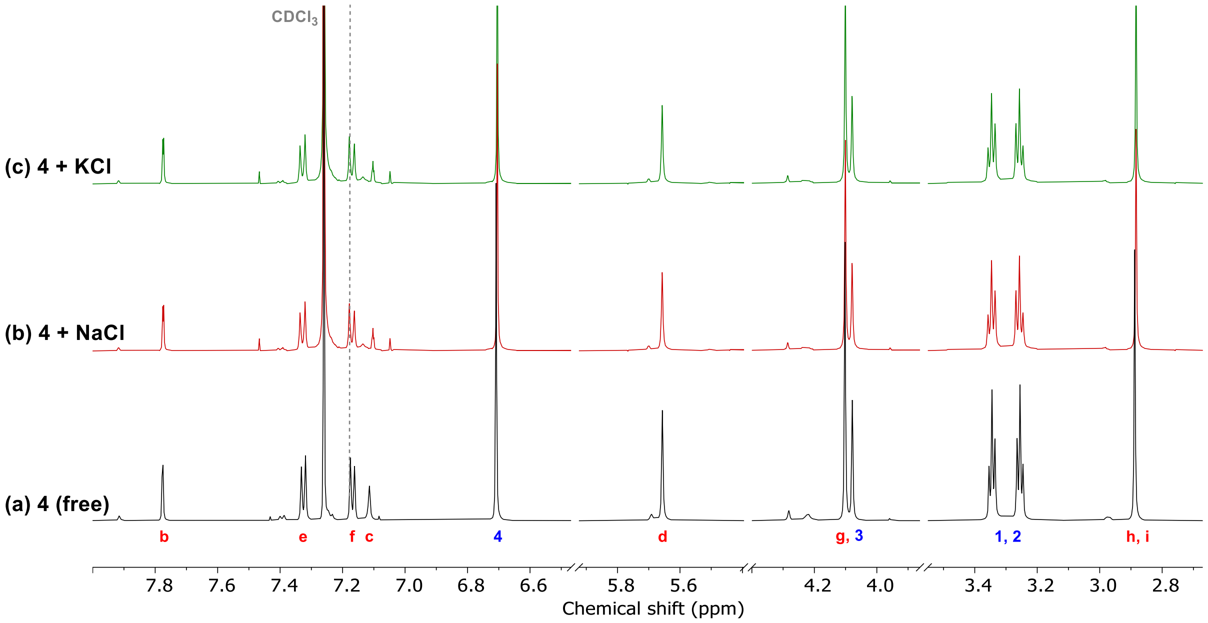

*Figure S47. Solid-liquid extraction studies of **4** with alkali metal chloride salts, showing truncated ^{1}H NMR spectra of: (a) free catenane receptor **4**, and post-extraction spectra of **4** following treatment with (b) 10 mg NaCl; (c) 10 mg KCl ($CDCl_3$, 298 K, 500 MHz).*

References

1. T. R. Chan, R. Hilgraf, K. B. Sharpless and V. V. Fokin, *Org. Lett.*, 2004, **6**, 2853-2855.
2. P.-N. Cheng, C.-F. Lin, Y.-H. Liu, C.-C. Lai, S.-M. Peng and S.-H. Chiu, *Org. Lett.*, 2006, **8**, 435-438.
3. H. M. Tay, Y. C. Tse, A. Docker, C. Gateley, A. L. Thompson, H. Kuhn, Z. Zhang and P. D. Beer, *Angew. Chem. Int. Ed.*, 2023, **62**, e202214785.
4. Y. C. Tse, A. Docker, Z. Zhang and P. D. Beer, *Chem. Commun.*, 2021, **57**, 4950-4953.
5. J. Cosier and A. M. Glazer, *Journal of Applied Crystallography*, 1986, **19**, 105-107.
6. L. Palatinus and G. Chapuis, *J. Appl. Crystallogr.*, 2007, **40**.
7. G. Sheldrick, *Acta Cryst. A*, 2015, **71**, 3-8.
8. R. Cooper, P. Betteridge, D. Watkin, K. Prout and J. Carruthers, *J. Appl. Crystallogr.*, 2003, **36**, 1487.
9. O. V. Dolomanov, L. J. Bourhis, R. J. Gildea, J. A. K. Howard and H. Puschmann, *J. Appl. Crystallogr.*, 2009, **42**, 339-341.
10. R. Cooper, A. Thompson and D. Watkin, *Journal of Applied Crystallography - J APPL CRYST*, 2010, **43**.
